# Examining the utility of process-focused data driven psychological networks for individualizing psychological treatment in chronic pain—A single case experiment testing the centrality hypothesis

**DOI:** 10.3389/fpsyg.2026.1809958

**Published:** 2026-04-21

**Authors:** Amani Lavefjord, Felicia T. A. Sundström, Loella Preihs, Alba Hammar, Sanna Forslund, Jakob Clason van de Leur, Saskia Scholten, Monica Buhrman, Lance M. McCracken

**Affiliations:** 1 Department of Psychology, Uppsala University, Uppsala, Sweden; 2 Department of Psychology, RPTU Kaiserslautern-Landau, Landau, Germany; 3 Centre for Biomedical Research in Epidemiology and Public Health (CIBERESP), Madrid, Spain

**Keywords:** acceptance and commitment therapy, ecological momentary assessment, idiographic, network analysis, process-based therapy, single case design

## Abstract

**Introduction:**

Idiographic network analysis, where associations between multiple nodes are estimated, can potentially guide choice of interventions in psychological treatment. In this single case experiment using ecological momentary assessment data to estimate continuous time network models, we aimed to test the so-called centrality hypothesis. We did so by comparing effects of interventions guided by the most central node to those guided by the least central node. We used the composite level psychological inflexibility processes lack of openness, lack of awareness, and lack of engagement, alongside an interference outcome, as network nodes. Effects on pain interference, motivation, and pain intensity were examined.

**Method:**

We employed a multiple baseline design across six participants. Therapists and participants were blinded to participants' treatment conditions. Baseline length and order of treatment phases were randomized.

**Results:**

Four participants had an overall treatment effect on pain interference, but it was generally not possible to discern that one particular phase was more beneficial than another. For three participants, the picture was somewhat clearer, indicating one of the treatment phases as more beneficial, although the results for these participants were not consistently in line with hypotheses. Retrospectively examining other potential guidance methods for these three participants, we saw a potential in discrete time contemporaneous network models.

**Discussion:**

Current results are not in line with previous assumptions or research on idiographic network models for treatment personalization, although the previous research in this area is scarce. Future research should investigate alternative network models or estimation choices to determine the potential utility of data driven idiographic networks.

## 1 Introduction

It is a cornerstone of psychological research to rely on group level data. At the same time, sole reliance on this approach is increasingly questioned, particularly when it comes to applying results from group level analysis to the individual person (Hayes et al., 2019; Lavefjord and Sundström, 2025; McCracken, 2023; Scholten et al., 2025a). General psychological models of how variables relate to each other might not be relevant for some or even many individuals, at least not as precisely as we have believed (Brose et al., 2010, 2015; Ciarrochi et al., 2024; Fisher et al., 2018; Gloster et al., 2024; Lowie and Verspoor, 2019; Molenaar and Campbell, 2009; Sahdra et al., 2024). A similar pattern seems to be present for people with chronic pain, who appear more heterogeneous than has been assumed, even within the same pain condition (Sundström et al., 2025). If models do not fit the individual, treatments based on those models are also unlikely to fit. This is the premise underlying process-based therapy, an approach focused on delivering interventions focused on person-specific processes of change (Hayes et al., 2019; Hofmann and Hayes, 2019).

Network analysis could help guide treatment by examining relationships among variables, also called nodes, within a system. One approach is the perceived casual network (PECAN) approach, where the networks represent one-time retrospective self-reports of how variables are perceived to affect each other (Klintwall et al., 2021). PECAN has been shown to be reliable and feasible (Andreasson et al., 2023; Kaariniemi et al., 2025; Klintwall et al., 2021; Reichert et al., 2025; Vogel et al., 2025), and recent work suggest potential clinical utility (Lavefjord et al., 2026). However, PECAN-guided interventions may not consistently improve all outcomes for all individuals (Lavefjord et al., 2026), indicating that alternative approaches to treatment guiding should also be explored. A key limitation of PECAN is its reliance on retrospective self-reports which are vulnerable to recall bias (Shiffman et al., 2008).

Data driven networks are instead based on repeated measurements (Bringmann, 2021; Epskamp et al., 2018; Epskamp, 2020; Robinaugh et al., 2020; Ryan and Hamaker, 2022), such as through the employment of ecological momentary assessment (EMA), where data are collected repeatedly over time in a person's real-life context (Shiffman et al., 2008). Multiple studies explore examples of person specific networks (e.g., Frumkin et al., 2021; Harris et al., 2025; Levinson et al., 2018, 2021; Ong et al., 2022; Scholten et al., 2022), and these networks are deemed useful by clients (Frumkin et al., 2021; Scholten et al., 2022). Recently, (Ong et al. 2025) conducted a study where person specific variables were included in data driven networks that were then used to complement an individual case conceptualization used for guiding a treatment plan (Ong et al., 2025).

Network *centrality* can also be used for treatment guiding. This hypothesis stipulates that intervening upon a central node in a network will affect the overall network more than intervening upon a less central node (Borsboom, 2017; Robinaugh et al., 2020). (Levinson et al. 2023) tested this in a previous study. Eating disorder related behaviors and experiences were included in a network, and interventions were selected that matched the most central nodes in each person's individual network. The results were promising, showing medium to large effects on many of the outcomes (Levinson et al., 2023), although there was no active control condition. In our recent PECAN study (Lavefjord et al., 2026), we included an active control in terms of interventions guided by the least central network node, and saw that the participants in general improved less in these conditions. It is not clear yet whether similar results would be found for data driven networks.

There is no consensus on the exact type of nodes to include in a network; for instance, whether to include specific problems, symptoms, or more functionally conceived contextual variables. In a PBT framework, it could be valuable to include psychological processes of change nodes. This is what we did in our recent PECAN study (Lavefjord et al., 2026). Specifically, we focused on acceptance and commitment therapy (ACT) process nodes. This was based on evidence that ACT is effective for increasing functioning and well-being in people suffering from persistent pain (Gloster et al., 2020; Lai et al., 2023), and because each process has a default intervention already coupled with it—making these processes useful for experimental testing of interventions matching particular network nodes. In support of this method, it has been shown that it is feasible to deliver specific ACT components separate from each other (Villatte et al., 2016).

In ACT, the aim is increasing psychological flexibility (PF), a general process shown to often mediate treatment effects (Hayes et al., 2022; McCracken, 2024; Murillo et al., 2022). The PF model specifies three overarching summary components, with two facets included per component (Hayes et al., 2013; McCracken, 2024). The overarching component of openness consists of the facet cognitive defusion, the ability to see your thoughts as no more or less than thoughts rather than something that has to govern your behavior, and the facet acceptance, the ability to allow experiences of thoughts, feelings, memories, or sensations, without resistance. The awareness component consists of present moment awareness, the ability to focus on what is relevant, and self as context, the ability to see yourself from a meta-perspective, knowing that you are more than your experiences or roles. The engagement component consists of values, in part including the ability to see clearly what is important in your life, and committed action, the ability to initiate, persist, and expand on striving for goals in line with your values (Hayes et al., 2013; McCracken, 2024). Lack of these skills is regarded as psychological inflexibility (PI; Hayes et al., 2013; McCracken, 2024). Lack of openness consists of fusion and experiential avoidance. Lack of awareness consists of lack of present moment awareness and self as content. Lack of engagement consists of lack of values and inaction.

This study in large replicates our recent PECAN study (Lavefjord et al., 2026), but with the aim of examining the utility of centrality in *data driven* individual networks as a source of guidance for treatment. We will do this by comparing the effects of intervention components based on the most and least central network nodes. We hypothesize that participants who receive interventions guided by the most central network node first will gain significant treatment effects upon this intervention, and no additional significant effects when subsequently receiving interventions guided by the least central network node. For those that instead receive the intervention guided by the least central node first, we realize that there could potentially be an effect due to receiving any intervention. If this happens, we hypothesize that additional significant effects will be seen subsequently when providing an intervention guided by the most central network node. Our primary outcome is pain interference, and a secondary outcome is motivation. We also explore potential effects on pain intensity, although this outcome does not always change from psychological treatment (Hughes et al., 2017). Lastly, we aim to retrospectively explore other potential methods for selecting the intervention to determine if any of these better align with the most beneficial outcomes for the participant.

## 2 Materials and methods

### 2.1 Procedure

We recruited participants through ads in Facebook groups and through e-mailing previous study participants having agreed to be informed about future studies. Following a link to REDCap (Harris et al., 2009, 2019), a secure online survey platform, participants were presented with participant information, provided informed consent, filled out background information and completed pre-study measures. Participants were then contacted by a therapist for scheduling an intake Zoom interview (Zoom Communications Inc, n.d.), using end-to-end encryption. During this meeting, eligibility was assessed by the therapist, individual treatment goals were collaboratively discussed, and the online treatment portal and daily assessment items were demonstrated and discussed.

Inclusion criteria were (1) age above 18 years old, (2) persistent or recurring pain for at least 3 months, (3) ability to speak, write, and read in Swedish to an extent enabling understanding of the treatment content and communication with the therapist, (4) willingness to be contacted throughout the study for receiving survey links, treatment portal updates, and, if needed, to be reminded by the therapist about assessments or treatment engagement, and (5) ability to follow the study protocol, including repeated daily assessments.

The study used a multiple baseline single case experimental design (SCED) across participants. After inclusion, participants began a baseline phase. In order to increase internal validity (Tanious and Onghena, 2019), baseline length was randomized to between 14 and 20 days. Participants were then exposed to two intervention phases. Each phase was by default 2 weeks long, but participants could ask for additional time for completion of sessions if needed. One of the phases delivered interventions predicted to be the *most* helpful, while interventions in the other were predicted to be the *least* helpful, based on total effect centrality in continuous time network analysis. The phase order was randomly determined. The website random.org was used both for assigning baseline length and order of intervention delivery.

Throughout all phases, participants responded to seven items five times a day, with one additional item at one daily measurement point. As such, the number of data points exceeded previous recommendations of at least four data points per SCED phase (Smith, 2012). Text message links to the REDCap daily assessments were sent at 8 AM, 11 AM, 2 PM, 5 PM, and 8 PM, using the add-on service (Twilio Inc, n.d.). Two reminders—one per hour—were sent out if the participant did not respond to the assessment. The participant was able to respond to the assessment during a 3-h window. After each phase, participants responded to some of the pre-study items again. At the end of intervention phases, they also evaluated goal attainment, and at the end of study, participants evaluated their general study experience. Both therapists and participants were blind to the resulting network and to the order of experiment conditions.

After treatment completion, therapists were briefed about the most and least central nodes in the participant's network. Therapists shared information regarding central nodes and treatment results with the participant during a final video meeting. Lastly, participants gained access to treatment content not included for them in the study, so that they could go through this content on their own if they wanted to do that.

The study was approved by the Swedish Ethical Review Authority (DNR: 2023-07383-01) and preregistered at open science framework (https://osf.io/5ftnc?view_only=d6944512f09e4cc5ae6f381a4d1eba2a).

### 2.2 Treatment

Participants received treatment methods from ACT. Treatment was delivered online, which has previously shown to be effective (Brown et al., 2016). The GDPR compatible platform Quenza (Quenza LLC, n.d.) was used to deliver treatment content and provided a secure chat for participants and therapists.

The ACT facets are naturally intertwined, but we aimed to separate the facets in order to create distinct intervention components. We followed a two-step procedure: First, we calculated composite variables of the three overarching components and included the composite variables as nodes in a first network together with the interference outcome variable, in order to find the most and least central node. Hence, treatment methods were matched to node centrality (e.g., the lack of openness node was matched to a treatment focus on increasing openness). Again, the delivery of the treatment method matching the most central and the least central node was randomly determined. Second, we adjusted the order of specific treatment interventions within the phases based on two subsequent network iterations. These second iteration networks comprised (a) only the specific facets of the *most* central overarching component together with the interference variable, and (b) only the specific facets of the *least* central overarching component together with the interference variable. The two networks were used to find the most and least central facets within the selected overarching components. The most central facet (e.g., experiential avoidance) was delivered first in the treatment phase based on the most central component (e.g., lack of openness), and the least central facet was delivered first in the treatment phase based on the least central component. This two-stage analysis was based on a recommendation to keep the number of variables low, as this has been shown to increase the validity of networks (Mansueto et al., 2023) while making certain that the beginning of each of the phases included the particular interventions potentially most (or least) helpful.

Treatment components were delivered as a combination of educational texts, case presentations, metaphors, and audio recorded experiential exercises. Participants were prompted to reflect upon the material and how it related to their lives and goals. Treatment was planned to be delivered at a pace of one facet (e.g., acceptance) per week, across two sessions, and the total treatment time was planned to 4 weeks. Participants were however able to ask for more time between sessions. Number of data points per participant and phase is found in Table 1. Participants applied their PF skills in practice between sessions, focused on applying PF skills in their lives while they worked toward their personal goals. See Table 2 for a description of the treatment in relation to network nodes, the same content as in our PECAN study (Lavefjord et al., 2026).

**Table 1:** Individual level participant characteristics.

ID	Age	Primary pain condition	Generalized pain	Pain duration	Previous treatment	Most central node	Least central node	Number of data points			Missing data points		
								Baseline	MCNI	LCNI	Baseline	MCNI	LCNI
1	56	Rheumatism	Yes	40 years	Yes	Lack of awareness (LPMA)	Lack of openness (Fusion)	74/15	94/19^*^	111/21	1/0	6/1	6/1
2	60	Leg and foot pain	Yes	Entire life	No	Lack of awareness (SAC)	Lack of openness (Fusion)	87/17	73/15^*^	76/15	3/1	2/0	0/0
3	41	Arthritis	Yes	15 years	Yes	Lack of awareness (LPMA)	Lack of openness (EA)	85/16	68/14^*^	68/13	5/2	2/0	1/1
4	43	Herniated disc, fibromyalgia, headache/migraine	Yes	17 years	Yes	Lack of awareness (LPMA)	Lack of engagement (Inaction)	77/15	58/10	49/11^*^	8/2	40/10	27/4
5	37	Headache/migraine	No	>10 years	Yes	Lack of openness (EA)	Lack of awareness (SAC)	74/15	104/21^*^	60/12	1/0	1/0	0/0
6	40	Ehlers-Danlos syndrome	Yes	25 years	Yes	Lack of engagement (LV)	Lack of openness (Fusion)	70/14	55/11	92/18^*^	0/0	5/1	3/1

MCNI, Most central node intervention phase; LCNI, Least central node intervention phase; LPMA, Lack of present moment awareness; SAC, Self as content; EA, Experiential avoidance; LV, Lack of values clarity; Number of data points and missing data points: all variables except motivation to the left/the motivation variable to the right. ^*^Indicating which phase came first for the participant.

**Table 2:** Network nodes and corresponding treatment content.

Network node	Corresponding treatment content
Lack of openness	Openness
Experiential avoidance	Acceptance—session 1: • Text based education about acceptance • Case-presentation to reflect upon: Peter with cancer avoiding his family—what should he be doing? • Audio-based skills training exercise: noticing sensations or emotions and opening up to them Acceptance—session 2: • Audio-based metaphors: the unwanted guest, and the struggle switch • Audio-based skills training exercise: noticing sensations or emotions and opening up to them in a long format and a shorter format
Fusion	Defusion—session 1: • Text based education about defusion • Audio-based metaphors: not thinking about a cup of tea • Audio-based skills training exercises: leaves on a stream, noticing one's thoughts • Verbal exercise: writing down thoughts with increasing levels of meta-awareness of them being thoughts Defusion—session 2: • Verbal exercise: writing down thoughts and giving them titles • Audio-based skills training exercise: viewing thoughts as if on a computer screen • Coming up with metaphors by oneself about thoughts if one is defused from them
Lack of awareness	Awareness
Lack of present moment awareness	Present moment awareness—session 1: • Text based education about present moment awareness • Audio-based metaphors: viewing the brain as a time machine • Case-presentation to reflect upon: Johan as a distracted parent—what are the consequences? • Audio-based skills training exercises: shifting attention, and a 20-min-long present moment awareness exercise Present moment awareness—session 2: • Several audio-based skills training exercises
Self as content	Self as content—session 1: • Text based education about self as context • Case-presentation to reflect upon: Mia as stuck in a particular social role that hinders her from doing what is important • Audio-based metaphor: consciousness as a flashlight • Audio-based skills training exercise: the self as something persistent Self as content—session 2: • Several audio-based metaphors: the self as a checkers board, and the self as the sky • Coming up with metaphors by oneself
Lack of engagement	Engagement
Lack of values clarity	Values clarity—session 1 • Text based education about values clarity • Audio-based metaphor: two kids in a car—one of them being restless about reaching destination, the other one finding meaning during the wait • Audio-based skills training exercises: 90-year-old birthday party • Verbal exercise: life compass Values clarity—session 2 • Case-presentation to reflect upon: Kim living according to others wishes—what are the consequences? • Verbal exercise: reflect upon life questions to find what your values are
Inaction	Committed action—session 1 • Text based education about committed action and strategies that can help for following through on commitments • Audio-based metaphor: going through a swamp • Verbal exercise: making your goals SMART • Case-presentation to reflect upon and applying systematic problem solving to: Anna struggling with following through on starting to run Committed action—session 2 • Continued education about strategies that can help for following through on commitments • Verbal exercise: weighing pros and cons for decision making

Participants received feedback and support on their reflections, homework assignments and questions when requested and after each session. This therapist support was delivered by licensed psychologists and graduate level students in the last term of a 5-year clinical psychology program. A supervisor who is a licensed psychologist and licensed psychotherapist with vast experience with delivering and supervising ACT, was available to the therapists upon request.

### 2.3 Measures

#### 2.3.1 Daily assessments

Pain interference, motivation, pain intensity, and each of the six PI processes were assessed with one item each, with responses measured on 0–100 VAS. See Table 3 for exact items. Higher scores were indicative of higher levels of the variable in question (i.e., higher pain interference, higher motivation, higher pain intensity, and higher levels of each PI process). While pain interference and inaction are similar in phrasing, the pain interference item was intended to measure more specific desired activities hindered by pain in particular, while inaction was broader in nature, encompassing anything that the participant had wanted to do but not succeeded in since the last measurement point, and not constricted to being related to the pain specifically.

**Table 3:** Daily assessment items.

Daily assessment variable	Item formulation
Pain interference	“How difficult was it for you to conduct the activity/activities that you wish to do?”
Motivation	“To what degree do you feel motivated to work toward your goals?”
Pain intensity	“Indicate your current pain on the scale below”
Experiential avoidance	“I avoided something unpleasant or that I thought would upset me”
Fusion	“I thought my thoughts were true and that caused problems for me”
Lack of present moment awareness	“I could not focus when I wanted to”
Self as content	“I thought that I could not change who I am”
Lack of values	“I did things that felt wrong or unimportant”
Inaction	“I could not get myself to begin or persist with something I wanted to do”

#### 2.3.2 Standard measures

##### 2.3.2.1 Demographics and pain background

Participants provided their details in terms of age, gender, relationship status, education status, work status, family origins, financial situation, and potential minority group status. They also indicated their pain condition(s), whether pain was generalized, medications for pain or mental health, pain duration, whether they had previously been through psychological treatment, and whether diagnosis had been made by a medical doctor.

##### 2.3.2.2 Interference variable from the Brief Pain Inventory

The primary outcome variable as measured in the daily assessments was complemented by administering seven interference items from the Brief Pain Inventory (BPI), a valid and reliable measure (Cleeland, 2009). The interference items were administered before baseline and after each phase and these assessed how pain affected mood, general activities, work, walking ability, sleep, relationship, and enjoyment of life the last 24 h. Items are scored on a 0–10 rating scale, with higher scores indicating more pain interference. We used a change score of 0.87 or more as indicative of meaningful change, based on conservative estimates of minimal important differences (Reed et al., 2024).

#### 2.3.3 Simple rating of perceived helpfulness

We included one pre-study question in layman terms about which of the ACT facets the participant thought would be most helpful for performing desired activities. We asked this at two timepoints; before and after baseline.

#### 2.3.4 Participant evaluation

After each intervention phase, participants were asked how much the received interventions had helped them in working toward their treatment goal and in achieving their treatment goal. At the end of the last intervention phase, participants were asked to evaluate the study in their own words and to indicate which treatment facet that had been most and least helpful to them.

### 2.4 Analyses

#### 2.4.1 Visual analysis

Visual analysis was conducted looking at stability of baseline, contrast between phases in means, trends, or variation, immediacy of change between phases, and overlap between phases, as recommended by (Wolfe et al. 2019). Following these recommendations (Wolfe et al., 2019), baseline stability was defined as a pattern enabling prediction of future patterns, phase contrast was defined as means, trends, or variation clearly differing from what was predicted by the baseline data, a high overlap was defined as more than 30 % overlap of datapoints, and immediacy was defined as a difference between the five last data points in a previous phase with the first five data points in a subsequent phase. We attempted to take all of these aspects into consideration when analyzing visual patterns, although, particular emphasis was put on phase contrast since immediacy is not necessarily theoretically expected, and since a high degree of overlap might stem from particular parts of the different phases (e.g., if there is no immediate change, the first part of a subsequent phase could overlap much with the previous phase) or from overall variability even in the presence of phase contrasts. Baseline stability was implicitly included in the phase contrast analyses, since differences in means, trends, or variability was determined in relation to the previous phase.

#### 2.4.2 Statistical analyses

All statistical analyses were conducted in R version 4.4.3 (R Core Team, 2025), using the packages ctnet (Ryan, 2025), ctsem (Driver et al., 2017), tibble (Müller and Wickham, 2023), qgraph (Epskamp et al., 2012), readr (Wickham et al., 2024a), dplyr (Wickham et al., 2023), pbapply (Solymos and Zawadzki, 2023), tidyr (Wickham et al., 2024b), zoo (Zeileis and Grothendieck, 2005), lubridate (Grolemund and Wickham, 2011), huge (Jiang et al., 2021), graphicalVAR (Epskamp, 2024), scan (Wilbert and Lüke, 2025), and scplot (Wilbert, 2025).

##### 2.4.2.1 Treatment guiding

Although discrete time (DT) vector autoregressive (VAR) network models have commonly been employed in psychological network studies, these come with assumptions of stationarity and equal spacing between assessment points (Ryan and Hamaker, 2022). We therefore used a continuous time (CT) VAR network approach for creating the idiographic networks for guiding treatment. For reproducibility, seed was set to 123 upon model estimation. Robustness testing in terms of replicating the analysis multiple times without set seed, indicated that the exact centrality values changed quite a lot from estimation to estimation. However, the order of node centrality in principle remained the same. Node centrality was based on total effect centrality (TEC), examining which PI process variable that, if decreased, would most likely tend to lead to decreases in remaining network variables. TEC was used because the treatment phases were short and this centrality index is expected to identify variables likely to exert a more instant effect on the network compared to central nodes indicated by indirect effect centrality (IEC; Ryan and Hamaker, 2022). Including posteriors in the centrality estimations, we specifically looked at the 50 % interval, and we set delta time to the median time interval between assessments for each participant.

##### 2.4.2.2 Treatment effect

Tau-U, a non-parametric single case statistic was used to evaluate treatment effect, as it adjusts for trend (Manolov and Moeyaert, 2017) and is minimally affected by autocorrelation (Barnard-Brak et al., 2021; Manolov and Moeyaert, 2017). We will specifically report results from the more conservative A vs. B + Trend B – Trend A, which adjusts for all phase trends. Generally, values below 0.2 are seen as small effects, 0.2–0.6 are seen as moderately high, 0.6–0.8 are seen as high, and values above 0.8 are seen as very high (Vannest and Ninci, 2015).

Missing data were interpolated using the built-in argument when building single case data frames in the scan package. Sensitivity analyses were carried out, where analyses were replicated with missing data excluded, and results were in principle very similar. The delivery of one overarching component, e.g., openness, that included two facets, e.g., acceptance and defusion, was treated as one single case phase. While it would be possible to also use the delivery of the separate specific facets as four phases, the data points were few for the motivation outcome for some participants, and, with generally fewer data points per such potential phase, the risk of carry over effects between phases was assessed as larger if shorter phases would have been used.

##### 2.4.2.3 Exploring alternative methods for treatment guiding

For a subset of the participants, namely those demonstrating clear beneficial effects in either of the phases, we also examined whether the intervention provided within that phase corresponded to interventions indicated to be useful from other methods for identifying the process that seems important to target. Specifically, we will descriptively present results from the pre- and post-baseline simpler assessments of which processes the participant thought would be most helpful, as well as mean baseline PI levels from daily measures. We also calculated Pearson correlations between PI processes and outcomes throughout the baseline.

Exploring other options within network analysis, we conducted additional CT-VAR network analysis, including nodes at the facet level and also exploring additional centrality indices, as well as looking at point estimates without including posteriors. We also explored CT-VAR centrality over wider ranges of delta times. We also conducted DT-VAR network analysis, applying normality transformation and detrending of the data. We looked at contemporaneous networks, both regularized and non-regularized, including nodes at the overarching summary component level as well as the facet level. We examined centrality in terms of expected influence, strength, betweenness, and closeness. The temporal DT-VAR networks were underpowered as indicated by a lack of edges in all temporal networks examined, and will therefore not be presented. In regards to both the CT-VAR and DT-VAR networks, we also examined which facet or summary component that had the strongest edge to the outcome variable.

#### 2.4.3 Participant evaluation

Participant evaluation of which facets that were retrospectively perceived as most helpful will be presented descriptively. Participant feedback on the study will also be summarized.

## 3 Results

### 3.1 Participant characteristics

**Figure 1:**
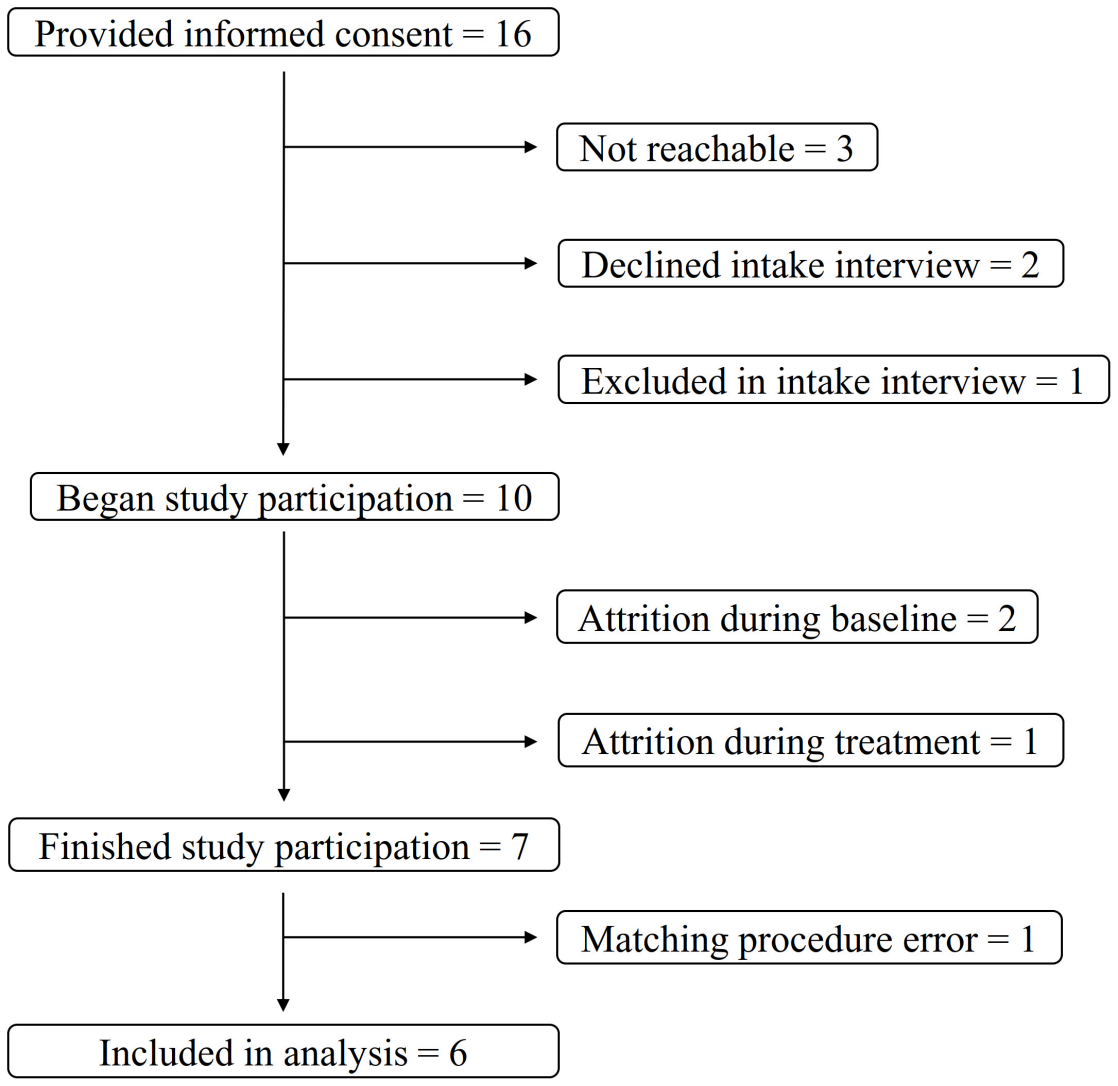
Flow chart of the number of participants.

Out of the 16 participants that provided informed consent for the study, data from six participants were analyzed for this paper. See Figure 1 for a participant flow chart. Most of these six were women, and one was non-binary. Their mean age was 46.17, ranging from 37 to 60, and all had Swedish origins. None identified as being part of a minority group. Half were in a relationship or married. Four participants were either full time or part time employed, one was a student, and one was seeking employment. Half of the participants had a university level education, one had completed vocational training, and two had graduated high school. Five participants described their income as sufficient, while one described it as good. Individual level participant characteristics can be found in Table 1.

### 3.2 Participant network centrality

**Figure 2:**
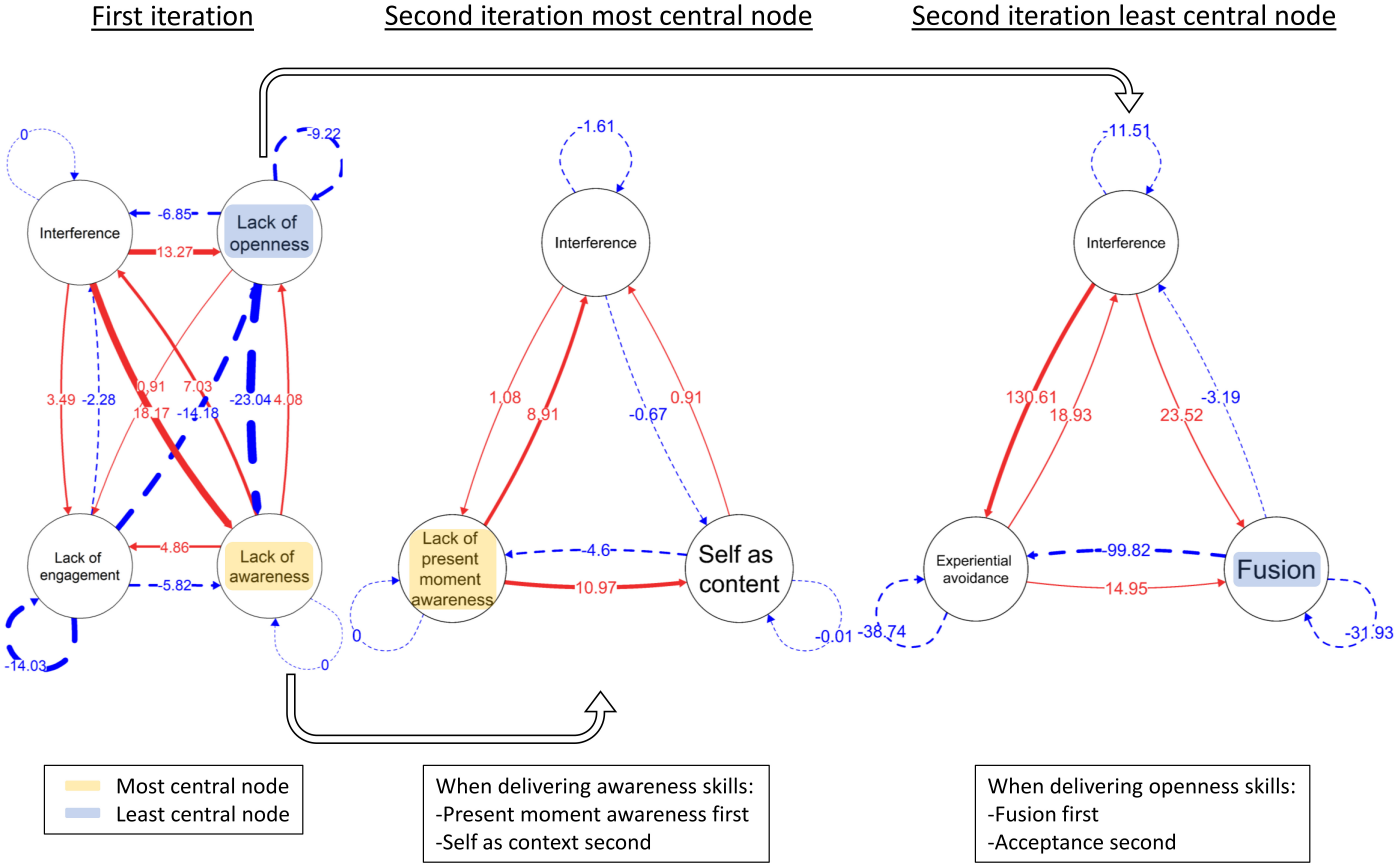
Network output for participant one. Participant one was randomized to receive the most central node intervention first. The figure highlights which node was the most central in the first iteration, and then illustrates the order of specific facets as determined by the second iterations.

Figure 2 illustrates the graphical network output for participant one. Network output for remaining participants can be found in Supplementary material. Most and least central nodes, as well as indication of whether the most central node intervention (MCNI) or the least central node intervention (LCNI) came first, are presented in Table 1. Exact centrality values are included in Supplementary material.

To exemplify the guidance procedure, we can see that for participant one, lack of awareness was the most central node. Within this overarching component, lack of present moment awareness was the most central in the second network iteration. As this participant was randomized to a treatment phase where they receive a most central node intervention first, they received skills related to awareness in the first treatment phase. Within this phase, they received present moment awareness skills before self as context skills. We also found lack of openness was the least central node. In the second iteration, fusion was the least central lack of openness node. Thus, in the second treatment phase, participant one received openness skills, where defusion skills was in focus followed by acceptance skills.

### 3.3 Descriptive statistics and visual analyses

Table 4 displays mean and standard deviations per phase and participant as well as results from the standard interference measures, indicating that five participants had a decrease in pain interference as measured by the BPI from end of baseline to end of treatment. However, it was not clear that the MCNI phase in particular yielded better results. One exception was participant six, who had a clear decrease in pain interference in the MCNI phase, but then had an increase again in the LCNI phase.

**Table 4:** Mean (and SD) for daily measures across participants and phases, and standard BPI pain interference scores across participants and phases.

ID	Mean daily interference			Mean daily motivation			Mean daily pain intensity			Standard BPI pain interference score			
	Baseline	MCNI	LCNI	Baseline	MCNI	LCNI	Baseline	MCNI	LCNI	Before baseline	End of baseline	End of phase 1	End of phase 2
1^*^	55.41 (23.92)	55.17 (19.34)	52.11 (17.03)	39.53 (20.15)	45.73 (17.44)	42.36 (15.23)	57.02 (13.76)	59.17 (12.38)	56.58 (12.24)	6.14	6.29	4.14^**^	2.86^**∧^
2^*^	18.54 (15.98)	8.59 (5.97)	2.47 (4.15)	89.69 (5.69)	96.73 (3.77)	99.53 (1.25)	18.56 (6.84)	16.36 (3.93)	18.61 (4.91)	3.71	6.00	1.86^**^	1.29^**∧^
3^*^	19.97 (20.76)	16.39 (16.46)	11.17 (16.90)	46.06 (21.23)	69.43 (23.83)	72.71 (19.40)	43.83 (11.83)	42.48 (11.36)	41.31 (9.92)	8.43	7.57	4.15^**^	5.86^**∧^
4	89.91 (8.23)	39.71 (36.23)	73.57 (29.86)	88.71 (25.51)	96.75 (4.88)	94.53 (7.78)	84.45 (8.44)	87.43 (7.77)	84.26 (6.27)	8.29	9.14	NA	7.57
5^*^	23.96 (25.28)	24.34 (22.97)	27.57 (23.81)	47.00 (28.71)	55.52 (20.99)	59.08 (12.39)	38.74 (12.09)	43.23 (9.49)	46.98 (9.91)	4.14	NA	3.57	2.14
6	47.51 (31.28)	37.00 (30.04)	28.81 (28.12)	32.43 (13.32)	40.50 (22.03)	49.87 (21.20)	44.79 (19.29)	33.62 (17.66)	30.38 (18.62)	4.00	NA	2.71^**^	6.29^**∧^

MCNI, Most central node intervention phase; LCNI, Least central node intervention phase. ^*^These participants received the MCNI phase first while the others received the LCNI phase first. ^**^Indicative of meaningful increase or decrease from the post-baseline measure if available and otherwise from the pre-baseline measure. ^∧^Indicative of meaningful increase or decrease from post-1.

**Figure 3:**
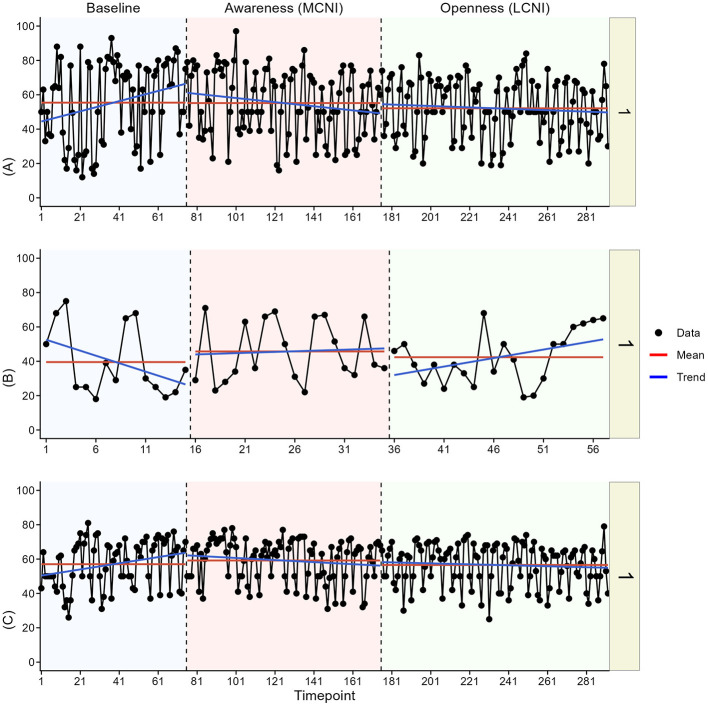
Daily scores for participant one on **(A)** pain interference, **(B)** motivation, and **(C)** pain intensity. MCNI, most central node intervention phase; LCNI, least central node intervention phase.

**Figure 4:**
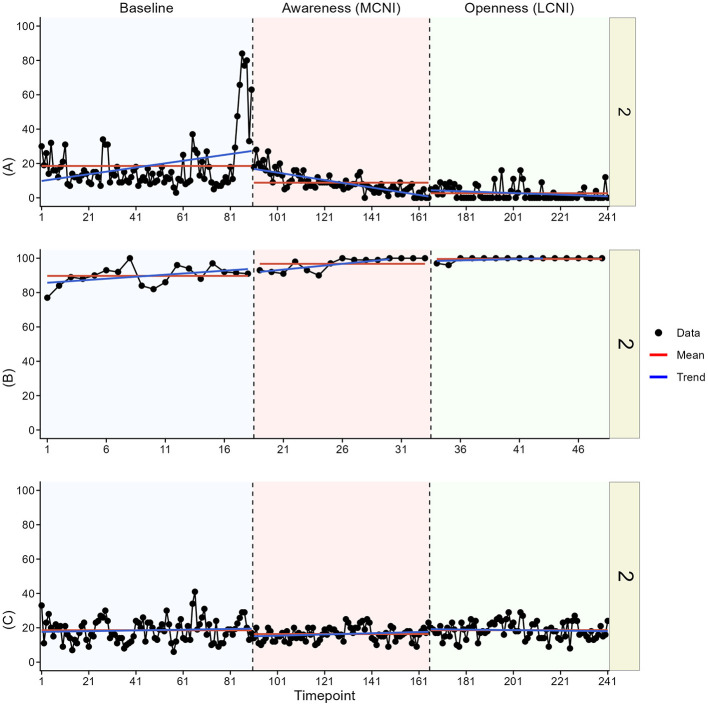
Daily scores for participant two on **(A)** pain interference, **(B)** motivation, and **(C)** pain intensity. MCNI, most central node intervention phase; LCNI, least central node intervention phase.

**Figure 5:**
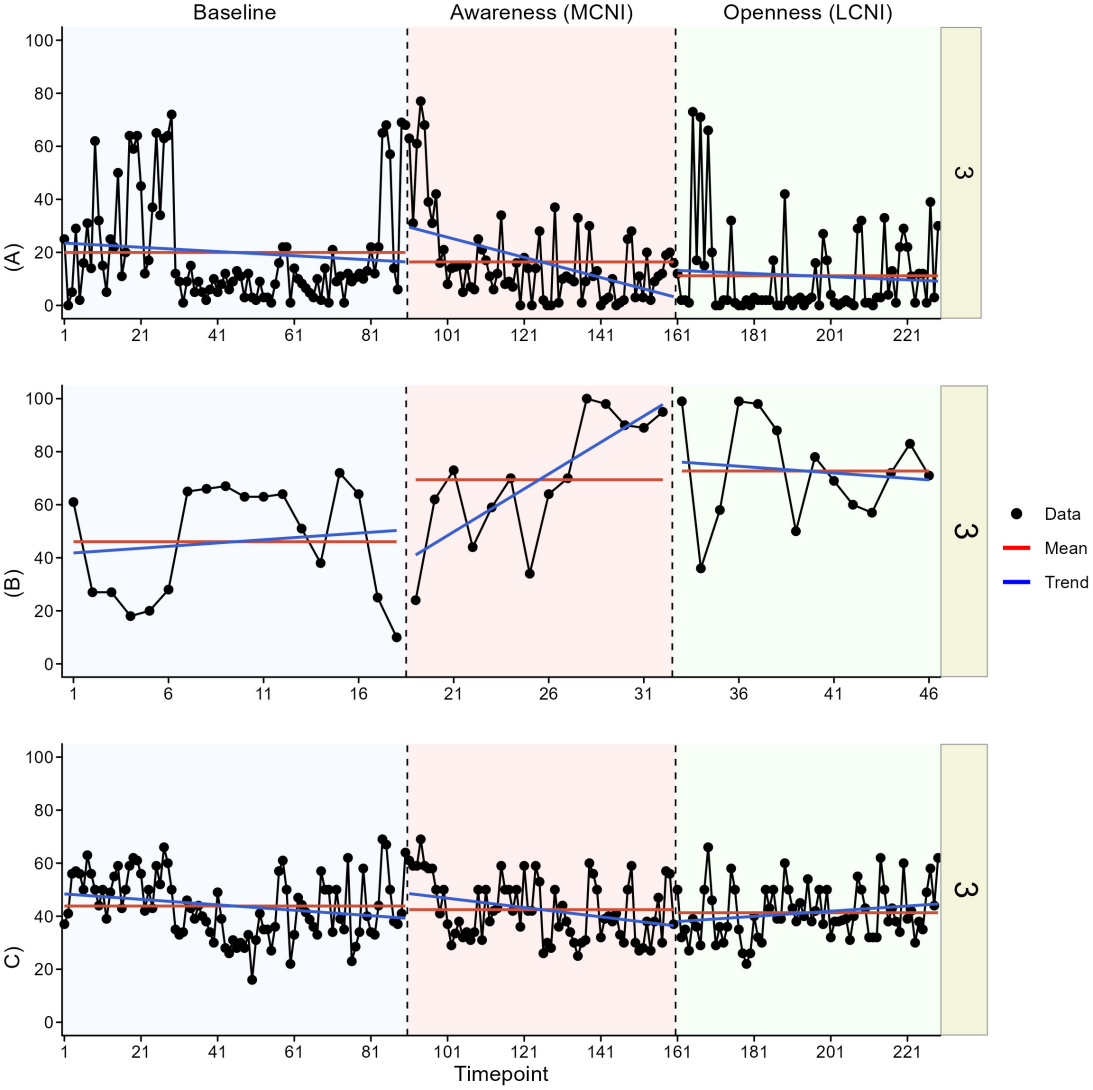
Daily scores for participant three on **(A)** pain interference, **(B)** motivation, and **(C)** pain intensity. LCNI, least central node intervention phase; MCNI, most central node intervention phase.

**Figure 6:**
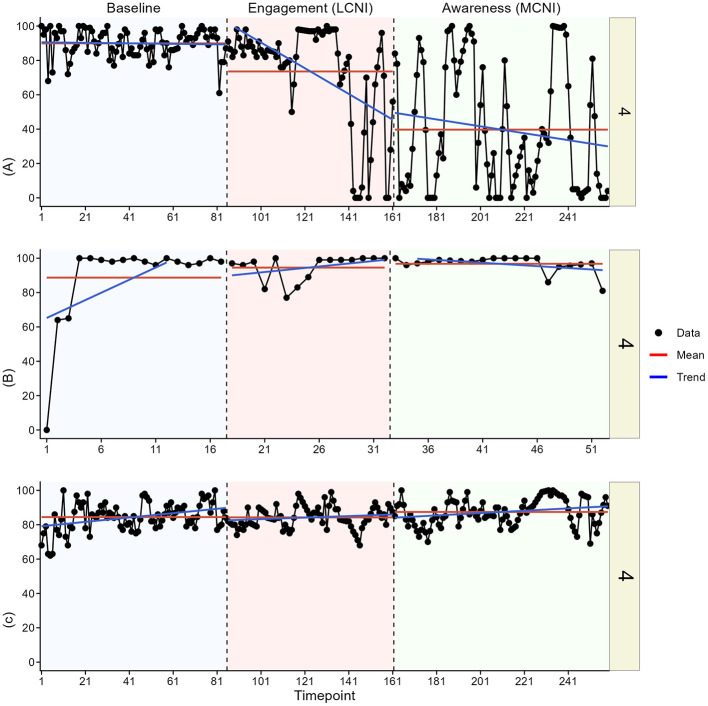
Daily scores for participant four on **(A)** pain interference, **(B)** motivation, and **(C)** pain intensity. LCNI, least central node intervention phase; MCNI, most central node intervention phase.

**Figure 7:**
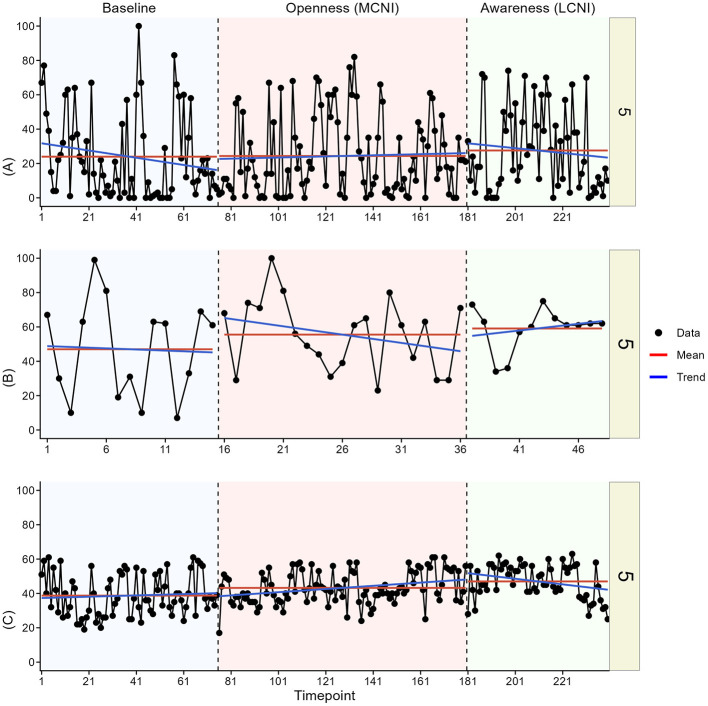
Daily scores for participant five on **(A)** pain interference, **(B)** motivation, and **(C)** pain intensity. LCNI, least central node intervention phase; MCNI, most central node intervention phase.

**Figure 8:**
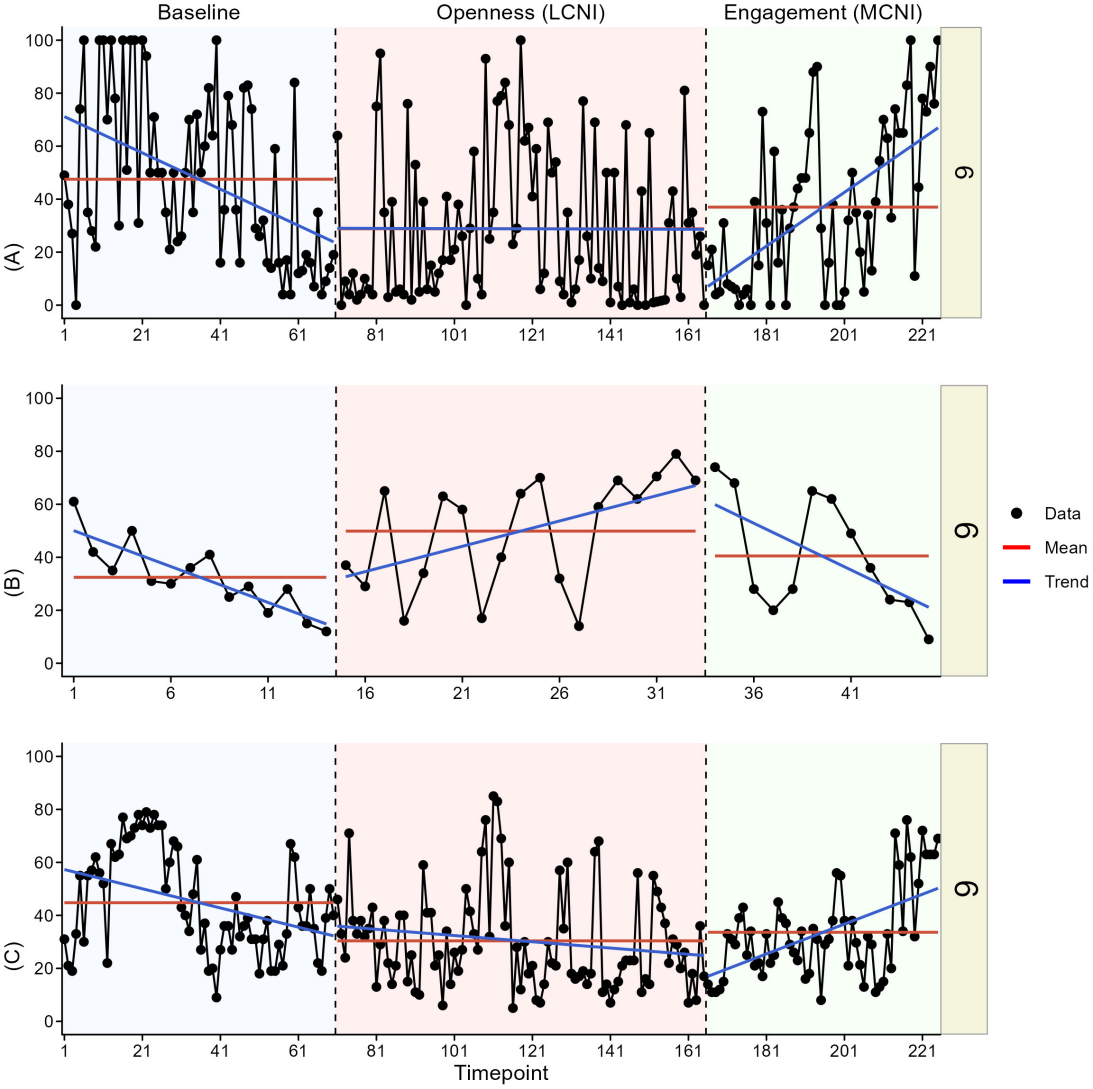
Daily scores for participant six on **(A)** pain interference, **(B)** motivation, and **(C)** pain intensity. LCNI, least central node intervention phase; MCNI, most central node intervention phase.

In the visual analysis, we most clearly see indications of meaningful change in at least one outcome for participant two, three, four, and six. See Figures 3–8 for visual graphs for each participant and each outcome.

#### 3.3.1 Participant 1

We saw no meaningful changes in pain interference or pain intensity between phases for participant one, other than potentially a change from an upward trend in baseline to downward trends in both intervention phases. The inverse was seen for motivation, with a downwards trend in baseline and upward trends in both intervention phases, but changes were subtle.

#### 3.3.2 Participant 2

For participant two, there was an immediate contrast in pain interference between baseline and the MCNI phase, and then an additional small contrast in mean and variation between the MCNI phase and the LCNI phase, but pain interference seemed to reach the low levels seen in the LCNI phase already by the end of the MCNI phase. Mean motivation was increased in the MCNI phase and then kept at these same high levels in the LCNI phase, although motivation was high already in baseline.

#### 3.3.3 Participant 3

For participant three, there was a cyclical pattern in pain interference throughout the entire study, but no clear indications of changes between phases. There were also no clear changes in pain intensity. For motivation, there was an immediate phase contrast between baseline and the MCNI phase, where motivation increased.

#### 3.3.4 Participant 4

Participant four showed a clear contrast in pain interference means between baseline and the LCNI phase and then again between the LCNI phase and the MCNI phase. The changes were however not immediate, as the decreases in pain interference were seen already at the end of the LCNI phase, continuing further during the MCNI phase. There were no clear changes in pain intensity and motivation, but we note that motivation increased sharply in the first 4 days of study participation.

#### 3.3.5 Participant 5

No meaningful changes were seen in pain interference or pain intensity for participant five. Motivation was generally slightly higher in both intervention phases compared to baseline.

#### 3.3.6 Participant 6

For participant six, there was a contrast in mean interference levels between phases, with the mean level being lowest in the LCNI phase, although with a downward baseline trend the contrast was not immediate. There was a slight mean decrease in pain intensity in the LCNI phase compared to baseline, but the intensity pattern looked somewhat cyclical throughout the entire study duration. Motivation was higher in the LCNI phase compared to the other phases, and there was also an upwards trend in contrast to the downwards trends in the other phases.

### 3.4 Tau-U

Details from the statistical analyses can be found in Table 5. In summary, the results did not generally support the hypothesis. When there were benefits from treatment, these did not seem tied mostly to the MCNI phase in particular.

**Table 5:** Tau-U results for participants receiving an intervention matching the most central CT network node first.

ID	Interference								
	Baseline-MCNI			Baseline-LCNI			MCNI-LCNI		
	Tau-U	SD_S	*z*	Tau-U	SD_S	*z*	Tau-U	SD_S	*z*
1	−0.09	773.47	−1.76	**−0.11**	**888.40**	**−2.31** ^*^	−0.05	1,065.94	−1.07
2	**−0.41**	**708.30**	**−7.62** ^**^	**−0.51**	**708.24**	**−9.40** ^**^	**−0.23**	**608.59**	**−4.01** ^**^
3	−0.05	677.16	−0.99	**−0.16**	**670.08**	**−2.92** ^**^	−0.07	547.46	−1.18
5	0.06	806.88	1.19	0.10	524.97	1.67	0.01	708.75	0.24
	**Baseline-LCNI**			**Baseline-MCNI**			**LCNI-MCNI**		
	**Tau-U**	**SD_S**	***z***	**Tau-U**	**SD_S**	***z***	**Tau-U**	**SD_S**	***z***
4	**−0.24**	**683.40**	**−4.41** ^**^	**−0.38**	**827.96**	**−7.48** ^**^	**−0.20**	**767.45**	**−3.90** ^**^
6	**−0.13**	**709.26**	**−2.43** ^*^	0.08	496.48	1.40	**0.14**	**645.89**	**2.54** ^*^
	**Motivation**								
	**Baseline-MCNI**			**Baseline-LCNI**			**MCNI-LCNI**		
	**Tau-U**	**SD_S**	***z***	**Tau-U**	**SD_S**	***z***	**Tau-U**	**SD_S**	***z***
1	0.20	70.31	1.65	0.20	76.23	1.74	−0.01	92.09	−0.07
2	**0.41**	**64.20**	**3.26** ^**^	**0.48**	**61.86**	**3.72** ^**^	0.23	49.39	1.64
3	**0.34**	**61.61**	**2.69** ^*^	**0.25**	**61.63**	**2.03** ^*^	−0.14	50.59	−1.01
5	0.03	73.31	0.26	0.15	47.84	1.07	0.14	64.41	1.16
	**Baseline-LCNI**			**Baseline-MCNI**			**LCNI-MCNI**		
	**Tau-U**	**SD_S**	***z***	**Tau-U**	**SD_S**	***z***	**Tau-U**	**SD_S**	***z***
4	0.09	60.51	0.68	−0.05	75.08	−0.43	−0.12	69.26	−0.98
6	**0.50**	**64.52**	**4.09** ^**^	0.17	45.32	1.21	**−0.34**	**58.80**	**−2.70** ^*^
	**Pain intensity**								
	**Baseline-MCNI**			**Baseline-LCNI**			**MCNI-LCNI**		
	**Tau-U**	**SD_S**	***z***	**Tau-U**	**SD_S**	***z***	**Tau-U**	**SD_S**	***z***
1	−0.03	772.60	−0.51	−0.08	887.80	−1.63	−0.08	1,066.50	−1.65
2	−0.07	708.23	−1.29	0.00	715.00	−0.01	0.09	619.88	1.56
3	−0.03	676.54	−0.51	0.02	670.25	0.34	0.08	547.73	1.29
5	**0.18**	**807.55**	**3.59** ^**^	**0.15**	**525.34**	**2.48** ^*^	0.00	708.98	0.03
	**Baseline-LCNI**			**Baseline-MCNI**			**LCNI-MCNI**		
	**Tau-U**	**SD_S**	***z***	**Tau-U**	**SD_S**	***z***	**Tau-U**	**SD_S**	***z***
4	−0.05	683.54	−0.96	**0.11**	**827.95**	**2.14** ^*^	**0.16**	**767.91**	**3.17** ^**^
6	**−0.22**	**709.42**	**−4.26** ^**^	−0.04	496.67	−0.63	**0.17**	**646.05**	**3.04** ^**^

MCNI, Most central node intervention phase; LCNI, Least central node intervention phase. Statistically significant results are in bold; ^*^p < 0.05; ^**^p < 0.001. SD_S, standard deviation of the count statistic used for calculating Tau-U.

### 3.5 Potential other sources of information for guiding treatment

As seen in the visual and statistical analyses, the results per participant were ambiguous. For instance, Tau-U results for participant four showed the MCNI phase as beneficial for pain interference, but worse for pain intensity. Results for participant six showed the LCNI phase as most beneficial for pain intensity but the MCNI phase as most beneficial for pain interference. We therefore moved on to look for participants who most clearly demonstrated consistent change across outcomes as well as across the visual analyses and the Tau-U analyses in any of the phases, in order to explore potential alternate sources of information for guiding treatments in these particular participants.

Specifically, we found that participant two showed decreased pain interference in the first delivered MCNI phase and then additionally in the subsequent LCNI phase, in both the visual analyses and Tau-U. Following the initial hypothesis, this additional change in the second intervention phase would indicate that the LCNI phase was the most critical. At the same time, the statistical effect is larger in the MCNI phase, and in the visual analysis the pain interference levels seen in the LCNI phase are reached already at the end of the MCNI phase, and motivation similarly increased in the MCNI phase. Putting all of the information together, the MCNI phase seemed to be the most beneficial one for participant two. While participant three did not have any statistically significant effects on pain interference, motivation increases in both the visual analysis and the Tau-U analysis during the MCNI phase, indicating the MCNI as the most beneficial. Lastly, we found that participant six consistently seemed to be better off in the LCNI phase, in both the visual analyses and the Tau-U.

Table 6 provides detailed information on baseline EMA measures and correlations. Table 7 presents which facets or summary components that were indicated as treatment targets based on pre-treatment participant simple assessments as well as alternative network-related guidance methods. Node centrality at different posteriors in the CT models are found in Supplementary material. We examined whether the indicated facets or overarching components matched which overarching component intervention that the selected participants got most help out of. Note that this is on an entirely explorative level and should not be seen as results *per se*, but rather as refined hypothesis generation.

**Table 6:** Exploring the highest baseline mean level PI process and correlations to outcomes.

Mean and correlations	EA	Fusion	LPMA	SAC	LV	Inaction
ID 2						
Mean	13.00	15.10	13.00	24.90	13.60	12.40
Correlation—pain interference	0.22	0.16	0.61	0.18	0.29	0.79
Correlation—motivation	−0.53	−0.21	−0.16	−0.10	−0.30	−0.50
Correlation—pan intensity	0.08	0.07	0.25	0.14	−0.04	0.08
ID 3						
Mean	9.04	7.66	17.60	1.56	6.20	11.10
Correlation—pain interference	0.44	0.32	0.75	0.22	0.42	0.61
Correlation—motivation	−0.54	−0.30	−0.70	−0.21	−0.43	−0.63
Correlation—pan intensity	0.16	0.07	0.43	0.05	0.14	0.29
ID 6						
Mean	14.20	48.20	19.30	47.90	40.60	49.40
Correlation—pain interference	0.15	0.50	0.21	0.52	0.69	0.79
Correlation—motivation	−0.36	−0.46	−0.10	−0.31	−0.37	−0.41
Correlation—pan intensity	0.14	0.43	0.12	0.38	0.32	0.24

EA, Experiential avoidance; LPMA, Lack of present moment awareness; SAC, Self as content; LV, Lack of values clarity.

**Table 7:** Exploring the facets in focus based on other potential matching procedures.

ID	Pre-baseline participant simple assessment	Post-baseline participant simple assessment	Strongest CT network-path to interference (4 variables)	Strongest CT network-path to interference (7 variables)	CT network TEC point estimate (4 variables)
2	Fusion	Self as content	Lack of engagement	Inaction	**Lack of awareness**
3	Inaction	Experiential avoidance	**Lack of awareness**	**Lack of present moment awareness**	**Lack of awareness**
6	**Experiential avoidance**	NA	Lack of engagement	Lack of values clarity	Lack of engagement
	**CT network TEC point estimate (7 variables)**	**CT network IEC point estimate (4 variables)**	**CT network IEC point estimate (7 variables)**	**CT smaller delta times TEC (4 variables)**	**CT smaller delta times IEC (4 variables)**
2	**Self as content**	Lack of engagement	Lack of values clarity	**Lack of awareness**/engagement	Lack of engagement
3	**Lack of present moment awareness**	Lack of openness	Lack of values clarity	**Lack of awareness**	Lack of openness
6	Lack of present moment awareness	**Lack of openness**	Lack of values clarity	Lack of engagement	**Lack of openness**
	**CT smaller delta times TEC (7 variables)**	**CT smaller delta times IEC (7 variables)**	**Strongest unpenalized DT contemporaneous network-path to interference (4 variables)**	**Strongest unpenalized DT contemporaneous network-path to interference (7 variables)**	**Strongest penalized DT contemporaneous network-path to interference (4 variables)**
2	**Self as content**/Lack of values	Experiential avoidance	**Lack of awareness**	**Lack of present moment awareness**	**Lack of awareness**
3	**Self as content**/Fusion	Experiential avoidance	**Lack of awareness**	**Lack of present moment awareness**	**Lack of awareness**
6	Lack of values	Lack of values clarity	Lack of engagement	Inaction	Lack of engagement
	**Strongest penalized DT contemporaneous network-path to interference (7 variables)**	**Penalized DT network strength and expected influence centrality (4 variables)**	**Penalized DT network betweenness centrality (4 variables)**	**Penalized DT network closeness centrality (4 variables)**	**Penalized DT network strength centrality (7 variables)**
2	**Lack of present moment awareness**	**Lack of awareness**	**Lack of awareness**	**Lack of awareness**	**Lack of present moment awareness**
3	**Lack of present moment awareness**	**Lack of awareness**	**Lack of awareness**	**Lack of awareness**	**Lack of present moment awareness**
6	Inaction	Lack of engagement	Lack of engagement	Lack of engagement	Self as content
	**Penalized DT network expected influence centrality (7 variables)**	**Penalized DT network betweenness centrality (7 variables)**	**Penalized DT network closeness centrality (7 variables)**	**Unpenalized DT network strength and expected influence centrality (4 variables)**	**Unpenalized DT network betweenness centrality (4 variables)**
2	**Lack of present moment awareness**	Inaction	**Lack of present moment awareness**	**Lack of awareness**	**Lack of awareness**
3	**Lack of present moment awareness**	NA^*^	**Lack of present moment awareness**	**Lack of awareness**	**Lack of awareness**
6	**Fusion**	Lack of values clarity	Lack of values clarity	Lack of engagement	**Lack of openness**/engagement
	**Unpenalized DT network closeness centrality (4 variables)**	**Unpenalized DT network expected influence and strength centrality (7 variables)**	**Unpenalized DT network betweenness centrality (7 variables)**	**Unpenalized DT network closeness centrality (7 variables)**	
2	**Lack of awareness**	**Lack of present moment awareness**	NA^*^	Inaction	
3	**Lack of awareness**	**Lack of present moment awareness**	NA^*^	**Lack of present moment awareness**	
6	**Lack of openness**/engagement	**Fusion**	Lack of values clarity	Lack of values clarity	

Bold = match with the overarching summary component that seemed to yield most preferable results for the participant. CT, continuous time; DT, discrete time; NA, Missing data. NA^*^ = No node that is clearly the most central.

We saw a match between node centrality and the most beneficial changes when exploring expected influence centrality in penalized contemporaneous DT-VAR networks when including seven variables, as well as in strength and expected influence centrality in unpenalized contemporaneous DT-VAR networks including seven variables. For these participants, the most specific central facet in these DT-VAR networks was one matching the overarching summary that was delivered when the participant had the most favorable outcome.

### 3.6 Participant evaluation

Participant evaluations were mostly positive, with interventions mostly described as meaningful and useful and the daily measures as a time for reflection. However, specific critiques that emerged related to a high burden with all assessments, a fast pace between interventions, and a lack of coherence in treatment due to the interventions being distinguished and given separately. Still, almost all participants were happy to have joined the study, although one participant reported that the treatment was not suitable for them. Self- assessed goal attainment after each phase and perceived most and least helpful facets can be found in Table 8. Seldomly did the perceived most helpful node match the most central network node.

**Table 8:** Self-assessed goal attainment after each phase and post treatment reports on perceived most and least helpful facets.

ID	Goal attainment after MCNI			Goal attainment after LCNI			Post report—most helpful	Post report—least helpful
	Getting started	Fulfilling	Mean	Getting started	Fulfilling	Mean		
1	70	39	54.50	81	62	71.50	Acceptance	Defusion^*^
2	29	71	50.00	61	78	69.50	Acceptance	Self as context
3	21	90	55.50	66	99	82.50	Acceptance	Present moment awareness
4	81	68	74.50	NA	NA	NA	NA	NA
5	47	70	58.50	33	76	54.50	Present moment awareness	Acceptance
6	76	58	67.00	62	100	81.00	Values^*^	Defusion^*^

MCNI, Most central node intervention phase; LCNI, Least central node intervention phase. ^*^Post reports of most and least helpful nodes matched the most and least central nodes. NA, missing data.

## 4 Discussion

The aim of this study was to examine the utility of node centrality in personalized data driven networks as guidance for selection of treatment components. We hypothesized that participants who would receive an intervention guided by the most central network node first would gain significant treatment effects upon this intervention, and no additional significant effects when subsequently receiving the intervention guided by the least central network node. For those that instead received the intervention guided by the least central node first, we hypothesized that it was possible that this could potentially yield treatment effects, but that additional significant effects would be seen subsequently when providing an intervention guided by the most central network node.

The present results were not in line with the hypotheses, as the most benefits were not clearly demonstrated during the phase that included interventions predicted as potentially most beneficial based on CT-VAR total effect centrality. While this is contrary to what is commonly assumed, it is potentially in line with voices questioning the importance of network centrality *per se* and advocating for other bases for interpretation of networks (Bringmann et al., 2019).

Network centrality can indeed be problematized regarding whether it constitutes a meaningful indicator of where to intervene. A statistically central node has many connections to other nodes, but may not be the most clinically meaningful node to address. For instance, in the current study, pain interference was defined as an outcome and only centrality values of PI processes were considered as potential treatment targets. A node may be central, but more difficult to change for a particular person, less accessible in therapy, or have less direct functional impact on the patient's daily life. If targeting such a process does not lead to noticeable improvements in functioning, this may reduce motivation or adherence and influence treatment outcomes. Additionally, the effectiveness of centrality-guided interventions may also depend on contextual factors such as treatment duration and intensity; it is possible that more time or therapeutic investment is needed for changes in central nodes to produce broader system-level effects. Of course, statistical centrality will also depend on which nodes that have been included in the network to begin with, and addition or subtraction of just a few other nodes could potentially change centrality. In this sense, centrality may represent one useful element in treatment decision-making rather than a sufficient criterion on its own.

In their recent proof of concept study, (Ong et al. 2025) did not rely on network *centrality* in particular when guiding treatment based on participant networks, so data driven networks might still hold promise outside of a centrality focus *per se*. On the other hand, our previous study, testing the centrality hypothesis in the context of PECAN rather than a data driven network (Lavefjord et al., 2026), instead did in fact indicate that network centrality may be a promising focus for treatment guidance. Further, (Levinson et al. 2023) previously also saw potential in using data driven network centrality as basis for treatment guidance.

If one continues to entertain the assumption that centrality matters, there could be other explanations for results obtained in the current study. First, we based the treatment guiding on TEC rather than IEI, even though IEI is hypothesized as the most important centrality index for treatment guiding (Ryan and Hamaker, 2022). Our rationale for selecting TEC instead, was the brief nature of the interventions and that multiple interventions followed one after the other, with the TEC being expected to be more rapidly affected by interventions. Still, it would have been interesting to replicate the study focusing on IEI instead. We did retrospectively examine whether IEI based treatment guiding could potentially have yielded the most beneficial effects, but such a retrospective examination would have been more thorough if all the possible overarching summary components had been presented and evaluated for effect. Second, we chose composite variables on the overarching summary component level as nodes in the networks, rather than using all facets as nodes. Third, we based the networks on a CT-VAR model rather than the so far more popularized method of DT-VAR networks.

We did retrospectively examine the utility of treatment guiding when estimating networks using more variables in CT-VAR networks, as well as DT-VAR networks based on both composite nodes and facet level nodes and using all the DT-VAR centrality indices. In these we consistently found that for the selected participants where we could clearly see beneficial results in one of the particular phases, there was a match in terms of the most beneficial outcomes occurring within a phase where one of the interventions corresponded to the most central node in the expected influence index looking at penalized contemporaneous DT-VAR networks. We found similar results when retrospectively focusing on strength centrality and expected influence centrality in unpenalized contemporaneous DT-VAR networks also including seven variables.

As a result of this study, we propose that future research prioritize further investigation of the network centrality hypothesis using methods where composite level nodes are used only after careful consideration—in our study, including variables corresponding to the specific facets rather than overarching summary component would potentially have been a better guiding principle. Further, looking at strength and expected influence in contemporaneous DT-VAR networks could hypothetically be more promising. However, using DT-VAR networks is not completely uncomplicated considering the need for equal spacing of assessments and stationary data. Here, the CT-VAR networks hold a large advantage, and we do also believe that these should continue to be examined for utility, potentially looking at IEI centrality and not using composite level nodes as we did here. In addition, in this study, we examined centrality at one fixed delta time set to the median time interval between assessments, essentially examining effects of one variable at one assessment point on the others at the subsequent assessment point. While a variable that is central in terms of affecting the network over time indeed would seem important for intervention, it is possible that more immediate relationships are a more important focus for intervention, as is indicated by the retrospective DT-VAR networks where the contemporaneous centrality indices showed promise. Of course, this could be dependent on the variables included in the network; it could be that more long-term temporal relations are more relevant for some variables, whereas shorter or immediate relations are more relevant for others. In this case, with processes that could potentially influence behavior in the short term, it seemed as though centrality based on more immediate relations would have been more relevant for guiding treatment.

We also urge researchers to continue examining the utility of PECAN, as this has shown clear potential. But again, with the potential sources of bias from participant retrospective recall, it would be ideal to also find data driven methods that could benefit participants. It is possible that data driven methods would suit some participants, whereas perceived causality methods would suit other participants better. Integrating PECAN and EMA data may also be a step forward (Scholten et al., 2025b).

Limitations to this study include the fast pace of intervention delivery, potentially hindering some individuals from finding the time and practice for applying the ACT skills for goal attainment. The limited length of each phase also increased the risks of carry-over effects between phases. Additional limitations include not delivering all ACT facets for effect evaluation, and the potential redundancy between the daily pain interference item and the daily inaction item—it would have been better to measure daily pain interference with more explicit idiographic measures, more clearly asking how pain had interfered with particular goal behaviors, rather than attempting to achieve this understanding in the intake video meeting. Many participants demonstrated a lot of variability in their baseline data, complicating analysis, and as mentioned, basing the networks on the overarching component level, hindered the assessment of facet level nodes. It is also worth noting that participants are homogenous in that all have had pain for a long time, no one identified as belonging to a minority group, all were women, and participants had a narrow age range. Although we do not intend to generalize the current results to an overall population, additional studies encompassing more heterogeneous samples can be of value if one wishes to move forward and explore generalizability. Further, we noted in robustness testing of network centrality, that exact centrality values differed a lot from estimation to estimation when seed was not set for reproducibility. This indicates that even with five assessments per day for at least 2 weeks, the number of data points may not have been sufficient for estimating robust networks. This study also has several strengths. It is the first study that directly tests the centrality hypothesis in data driven networks by including a control condition focused on interventions guided by the least central node, and it does so by using randomization of both baseline length and phase order, as well as utilizing double blinding regarding the most and least central nodes.

While we did not find support for our initial hypotheses, this study has generated new hypotheses for future investigation, and brings us closer to an understanding of how, if at all possible, to effectively use network analysis to provide guidance in individual process-based treatment.

## Data Availability

The raw data supporting the conclusions of this article will be made available by the authors, without undue reservation.
