# Supplementary material for: Examining the utility of process-focused data driven psychological networks for individualizing psychological treatment in chronic pain—A single case experiment testing the centrality hypothesis
Source: Front Psychol. 2026 Apr 21;17:1809958. doi: 10.3389/fpsyg.2026.1809958 (PMC13139109; doi:10.3389/fpsyg.2026.1809958)
Supplement: Supplementary file 1 [file Table_1.docx]

**Supplementary Material**

**Figure S1a.** First iteration of network output for participant two


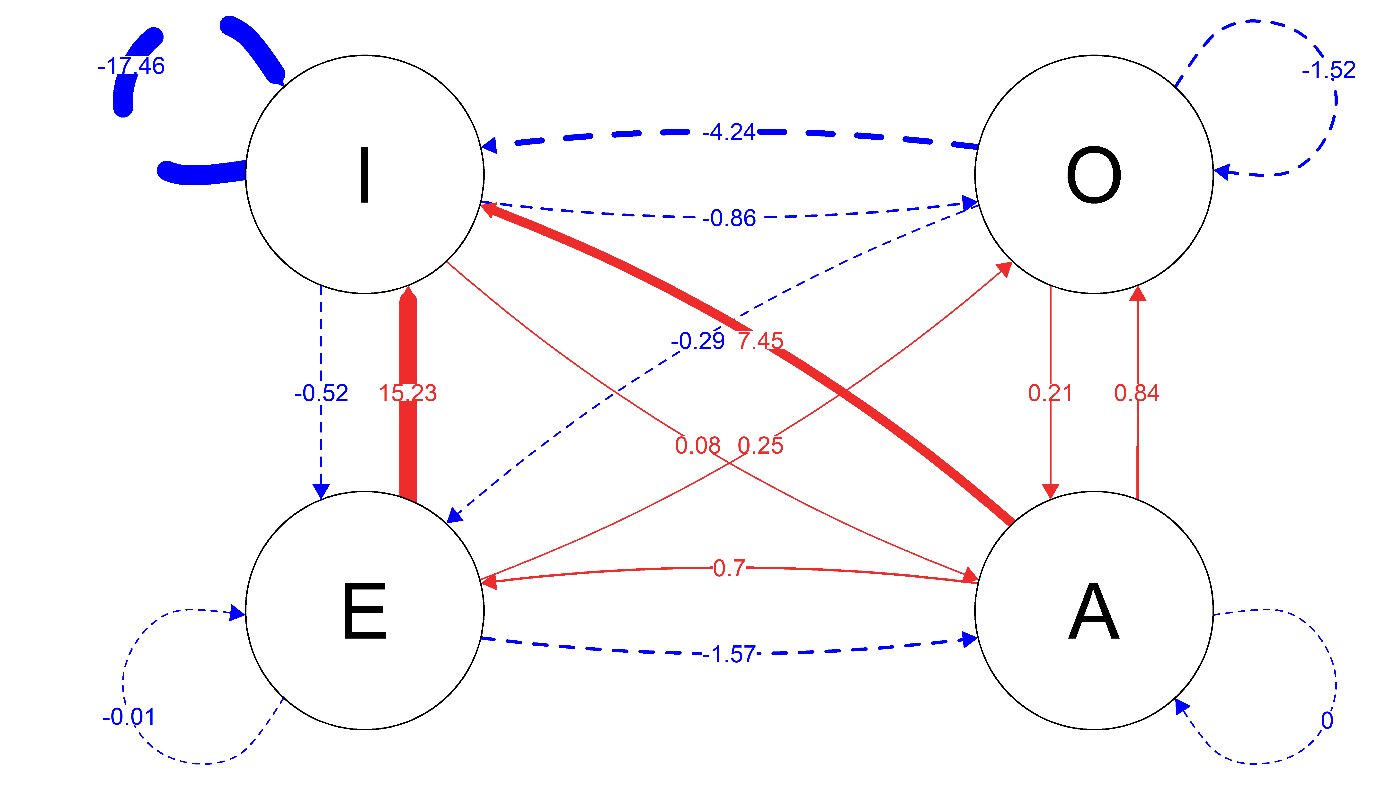


**Figure S1b.** Second iteration of most central network node for participant two


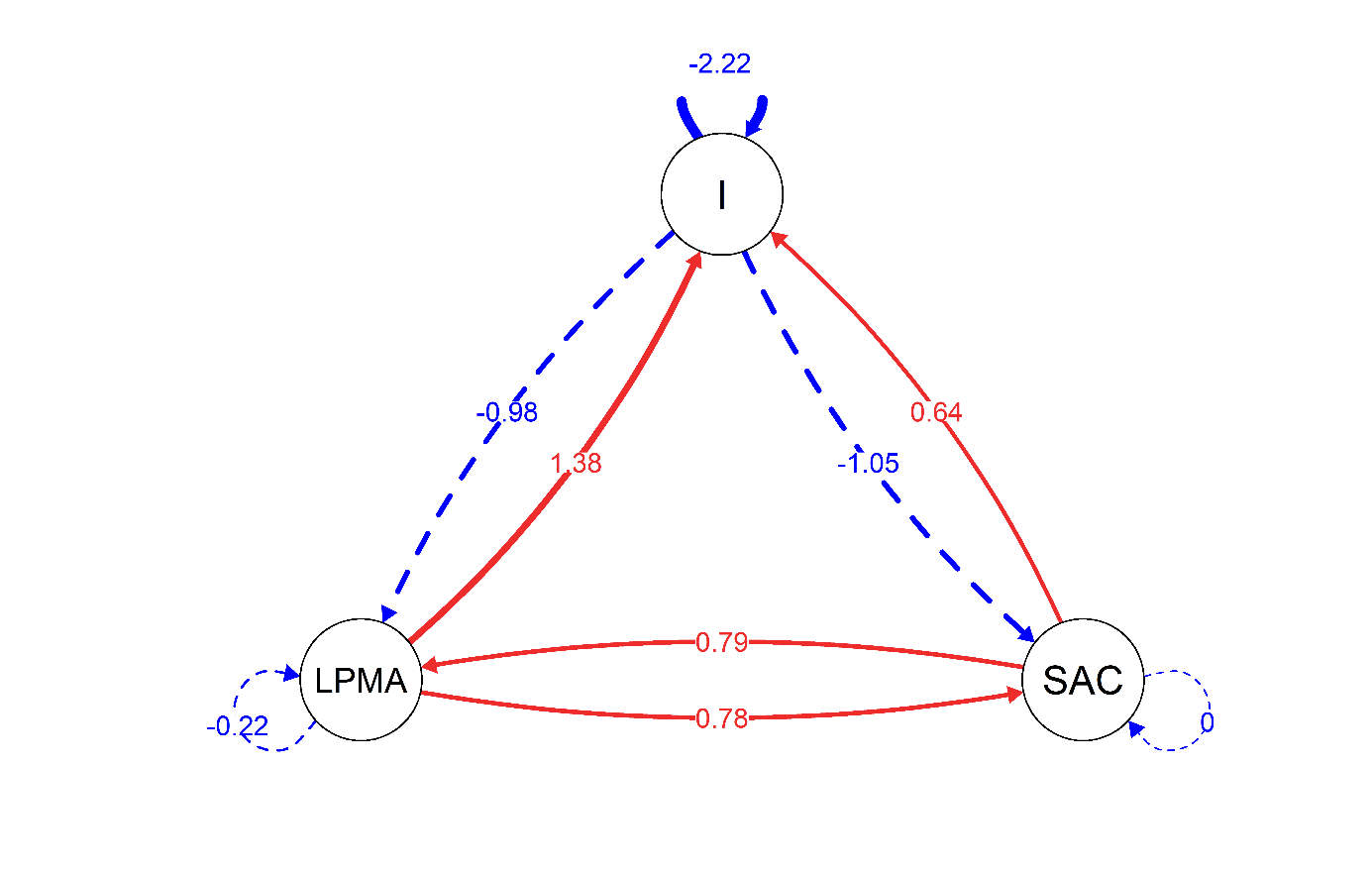


**Figure S1c.** Second iteration of least central network node for participant two


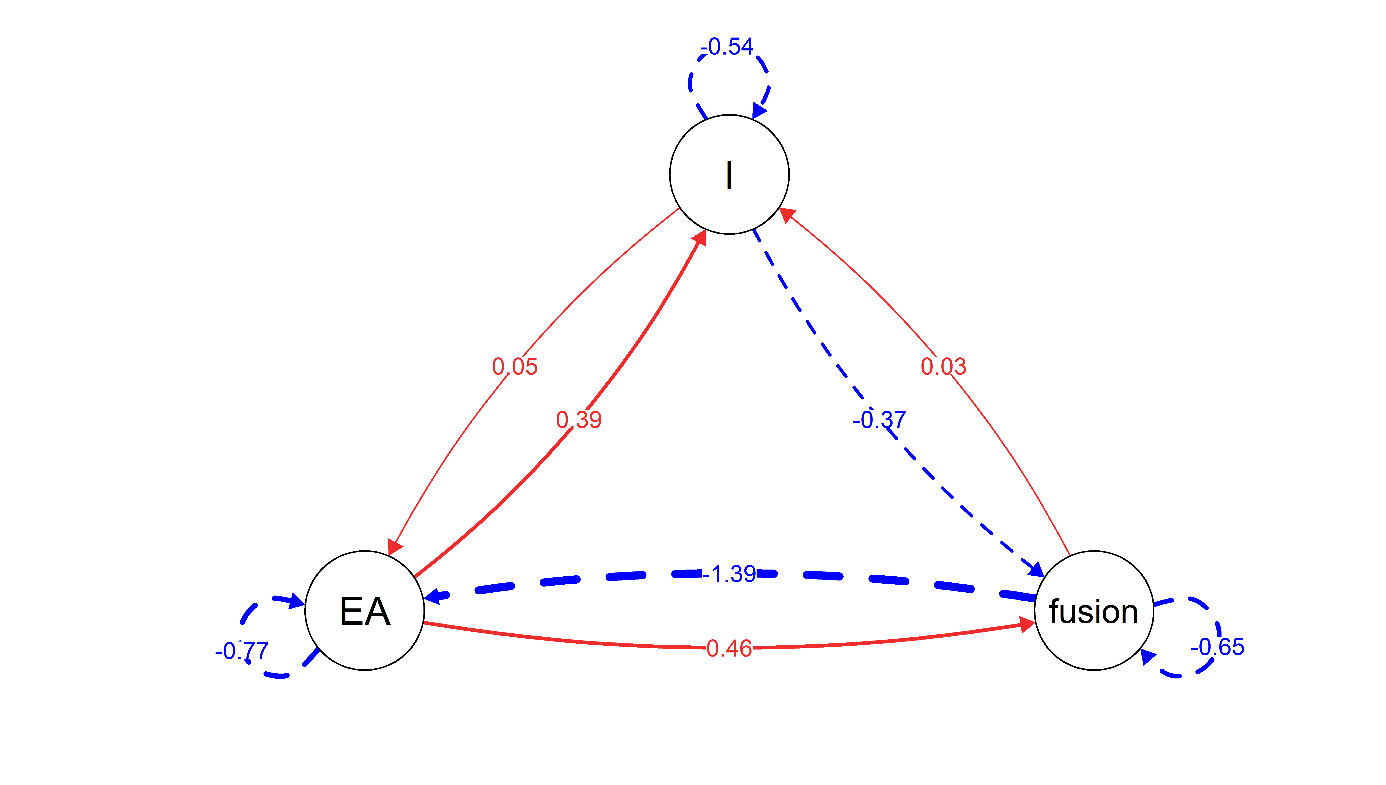


*Note.* I = Interference. O = Lack of openness. A = Lack of awareness. E = Lack of engagement. EA = Experiential avoidance

**Figure S2a.** First iteration of network output for participant three


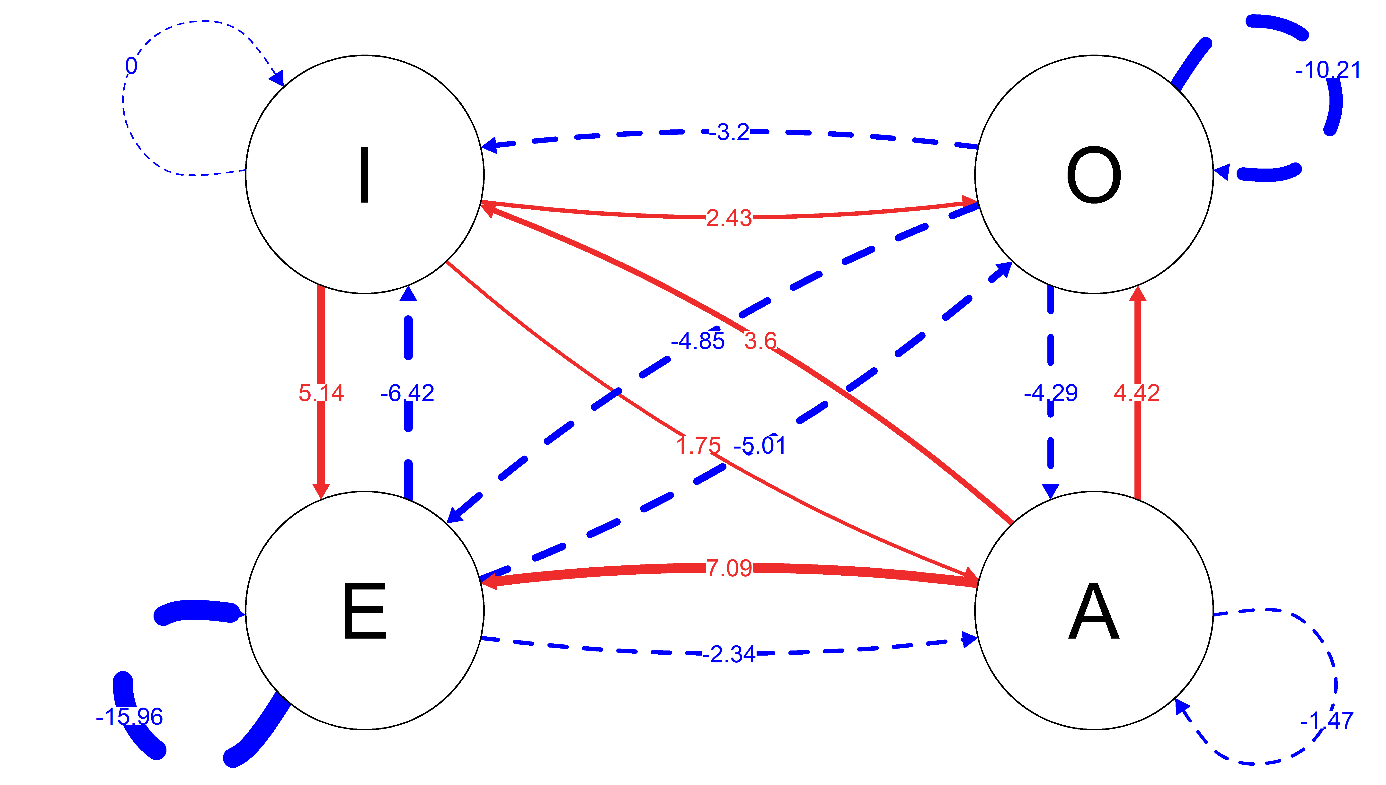


**Figure S2b.** Second iteration of most central network node for participant three


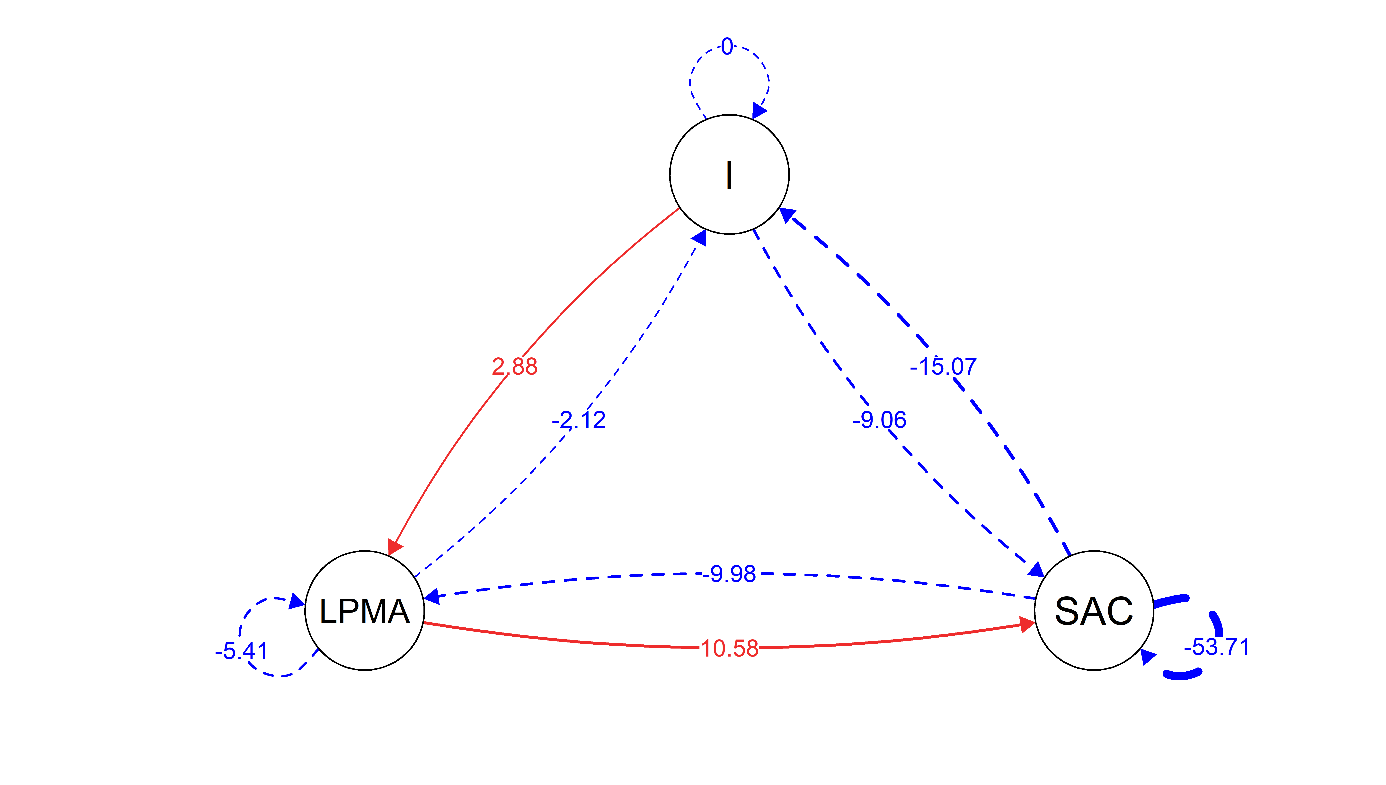


**Figure S2c.** Second iteration of least central network node for participant three


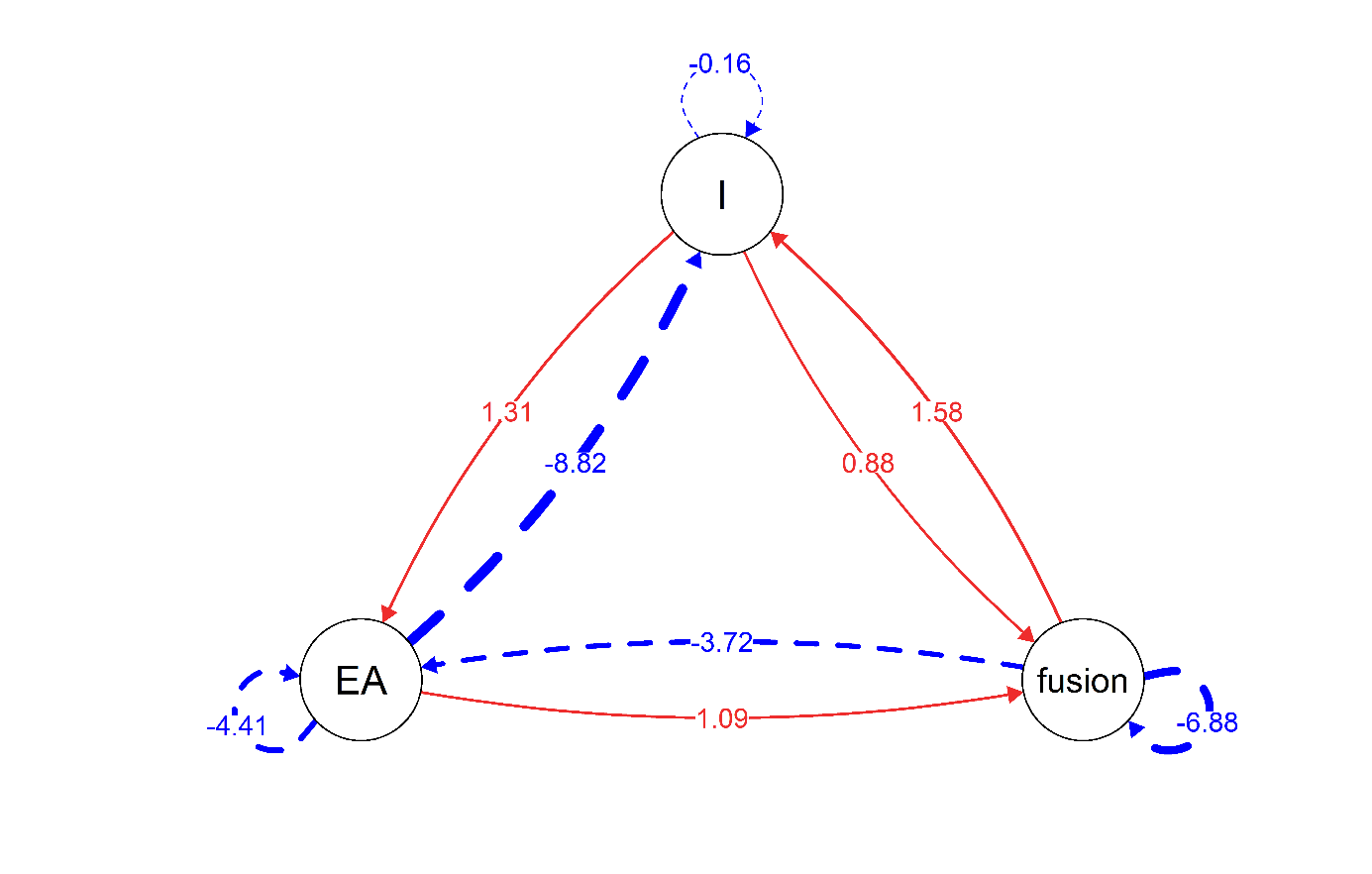


*Note.* I = Interference. O = Lack of openness. A = Lack of awareness. E = Lack of engagement. LPMA = Lack of present moment awareness. SAC = Self as content. EA = Experiential avoidance.

**Figure S3a.** First iteration of network output for participant four


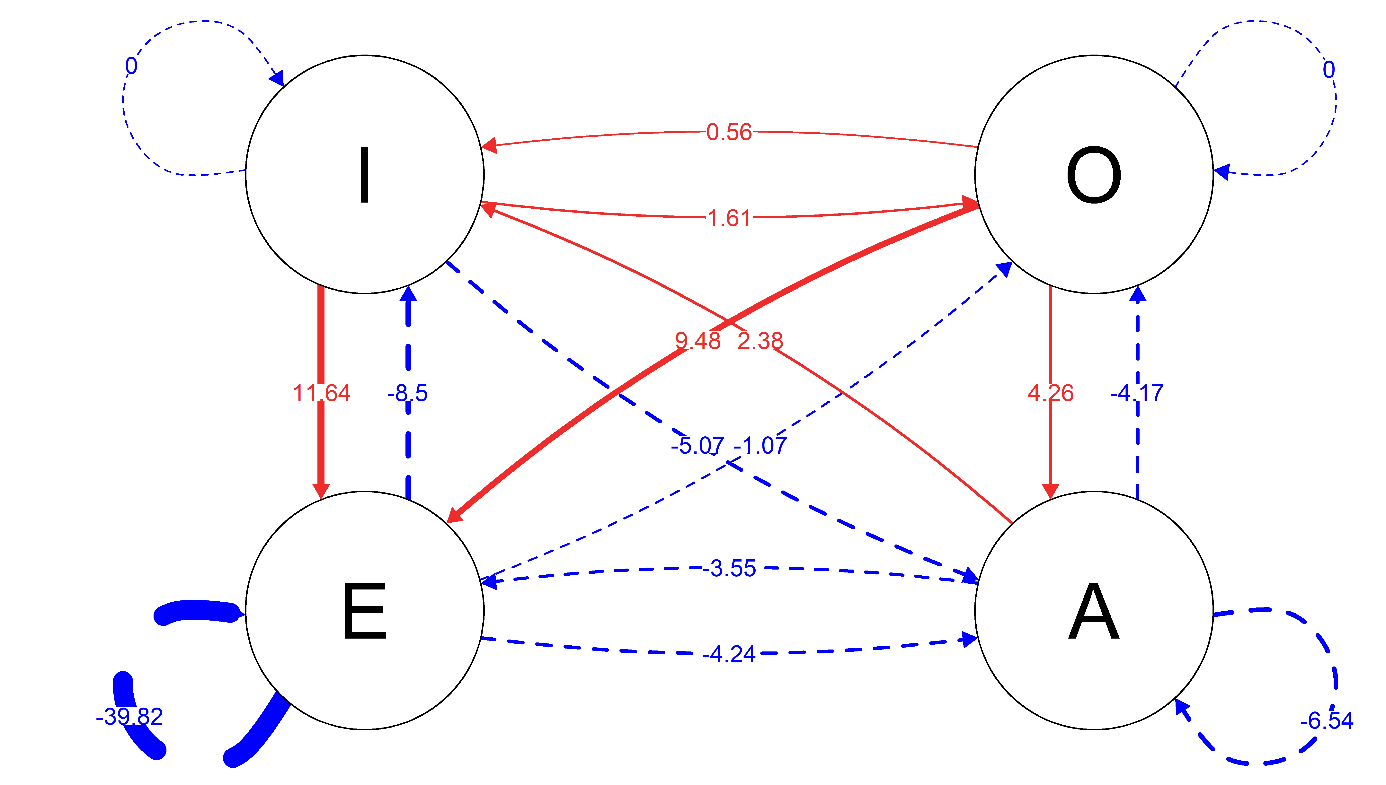


**Figure S3b.** Second iteration of most central network node for participant four


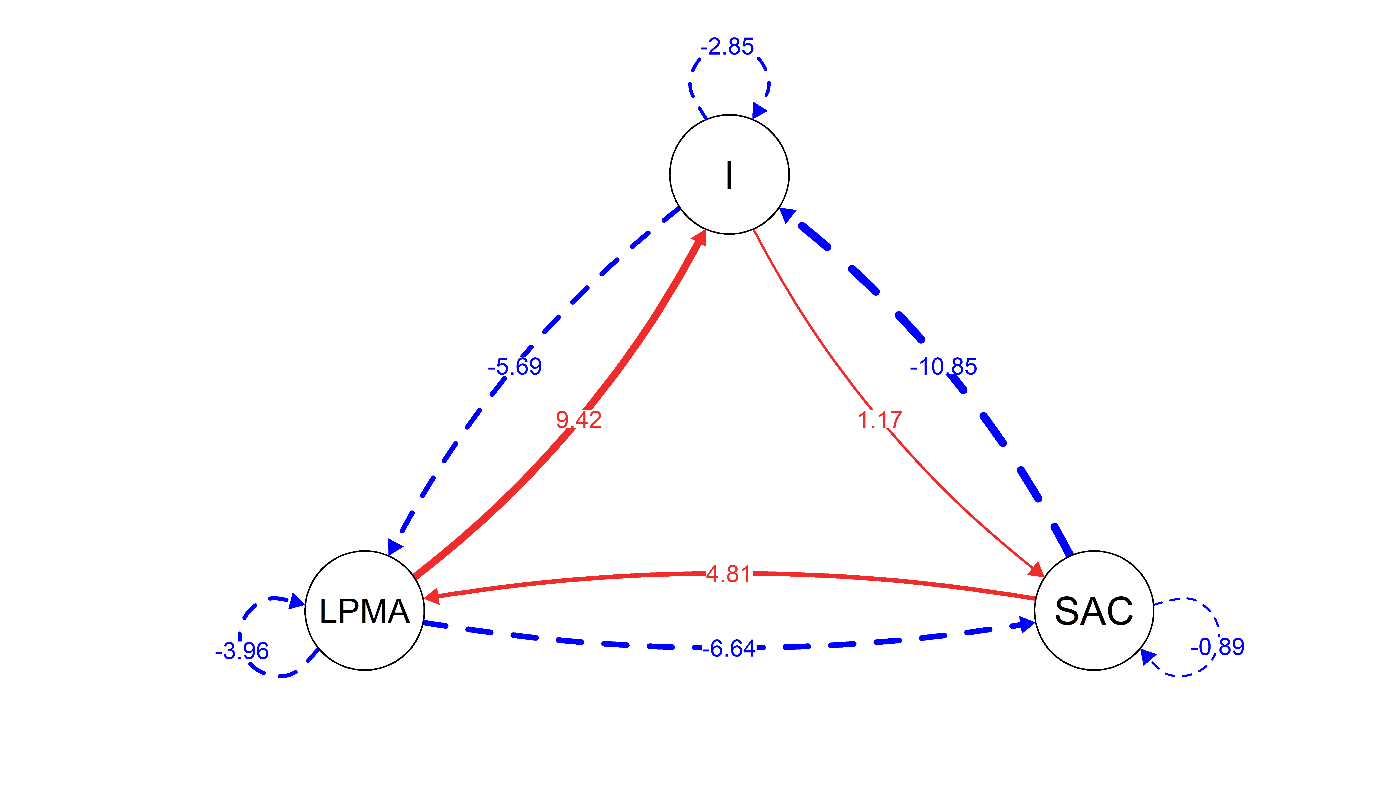


**Figure S3c.** Second iteration of least central network node for participant four


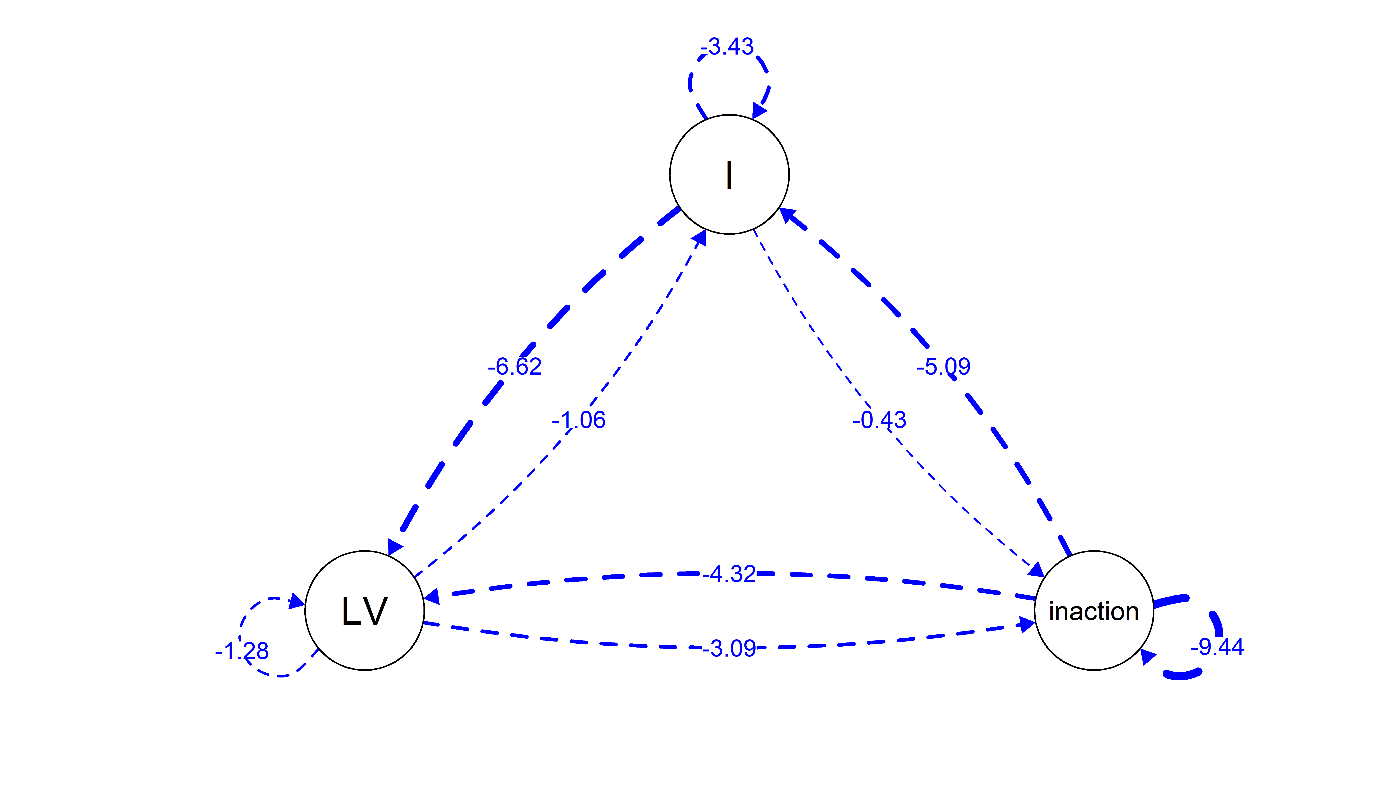


*Note.* I = Interference. O = Lack of openness. A = Lack of awareness. E = Lack of engagement. LPMA = Lack of present moment awareness. SAC = Self as content. LV = Lack of values clarity.

**Figure S4a.** First iteration of network output for participant five


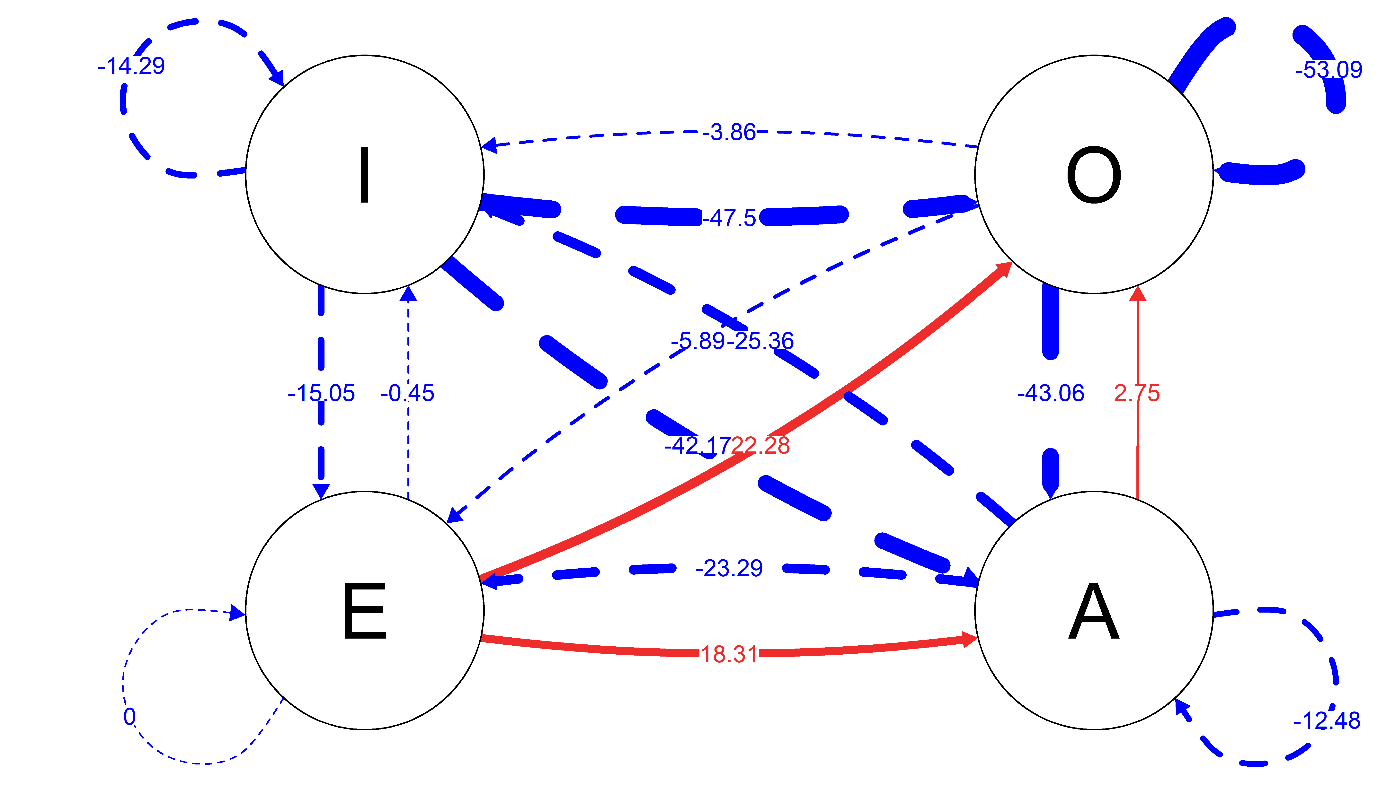


**Figure S4b.** Second iteration of most central network node for participant five


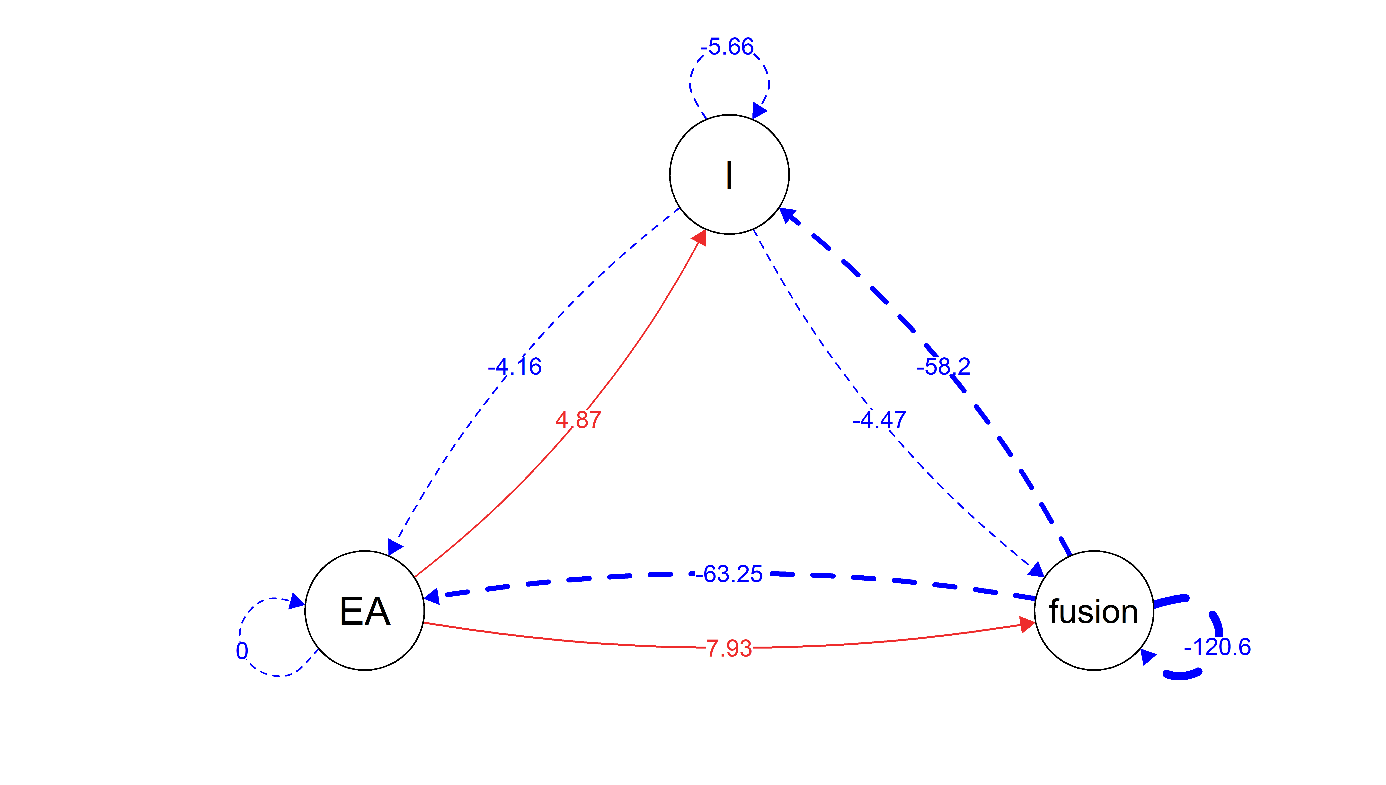


**Figure S4c.** Second iteration of least central network node for participant five


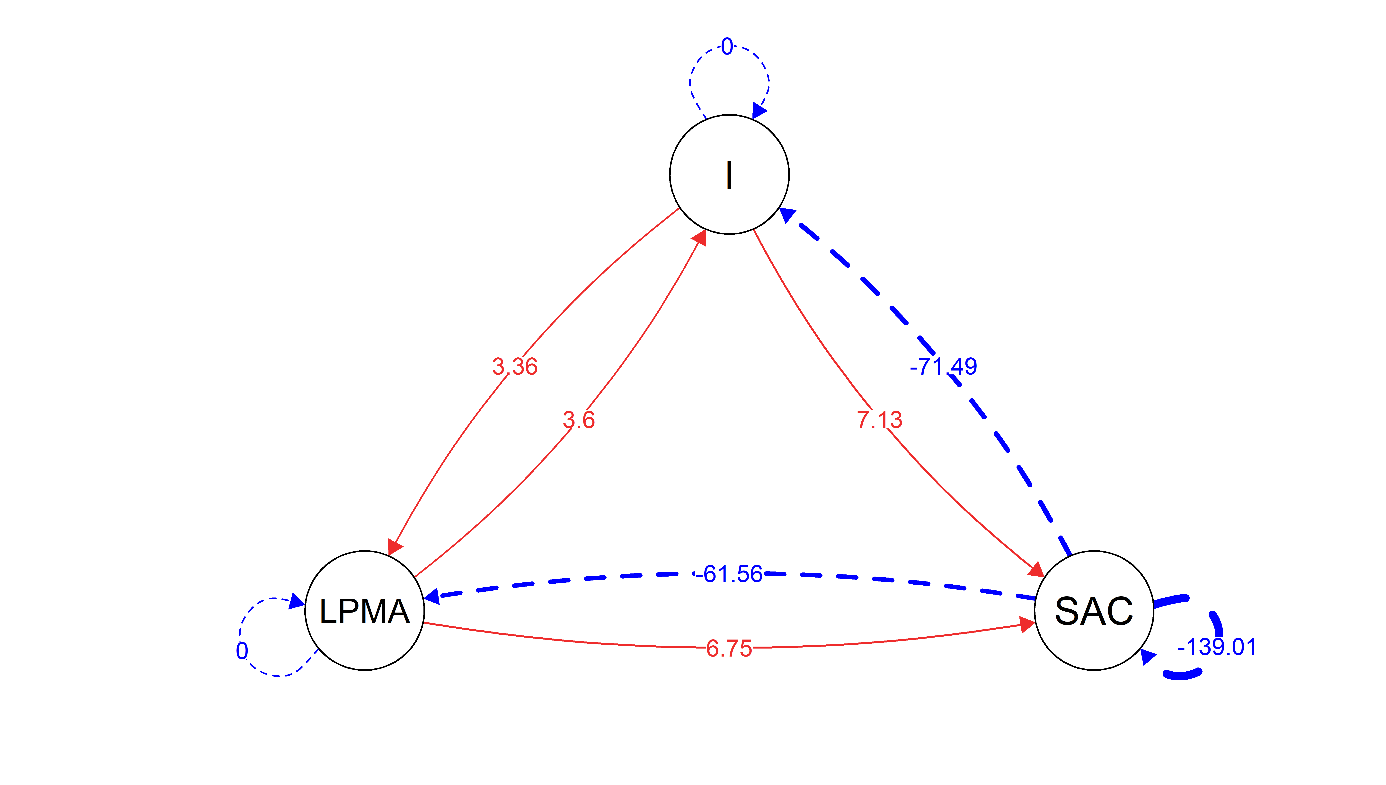


*Note.* I = Interference. O = Lack of openness. A = Lack of awareness. E = Lack of engagement. EA = Experiential avoidance. LPMA = Lack of present moment awareness.

**Figure S5a.** First iteration of network output for participant six


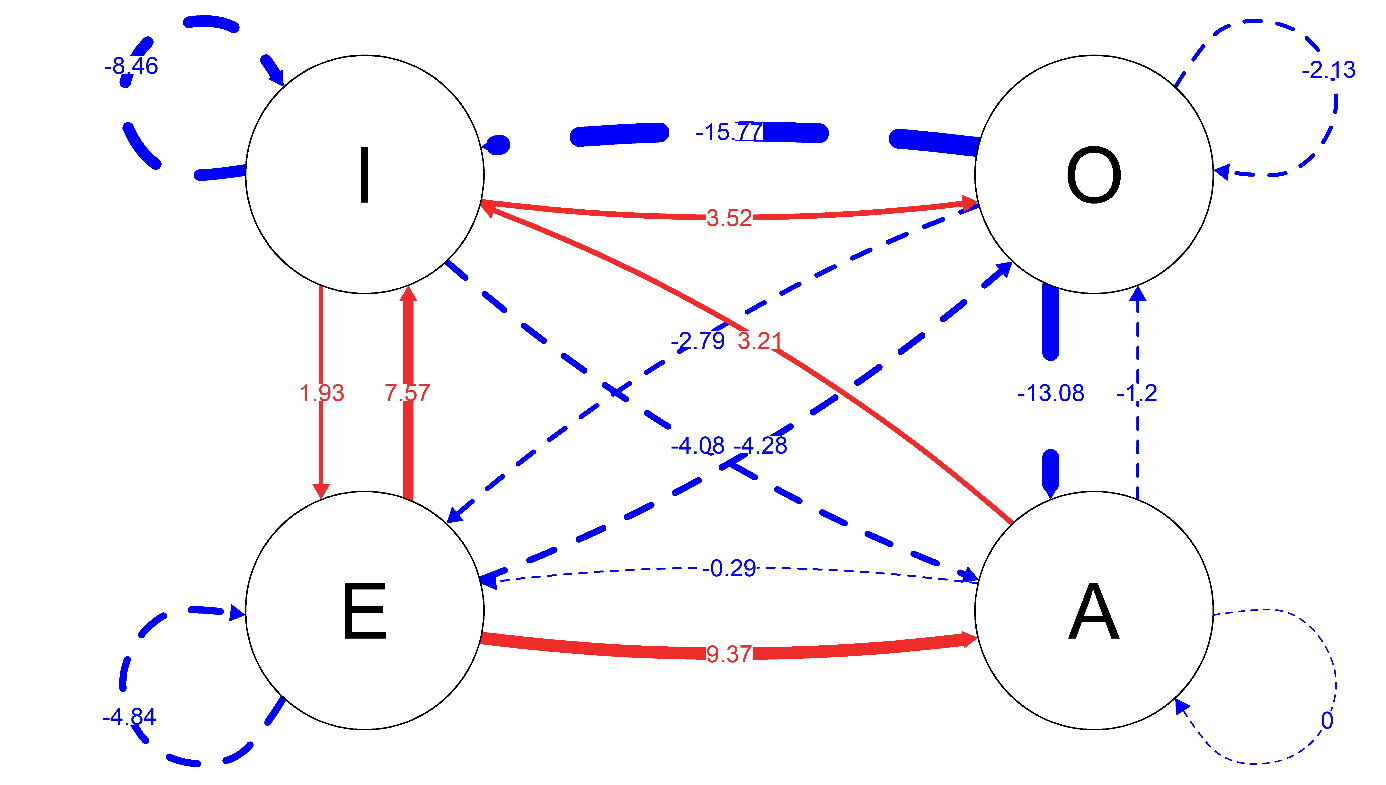


**Figure S5b.** Second iteration of most central network node for participant six


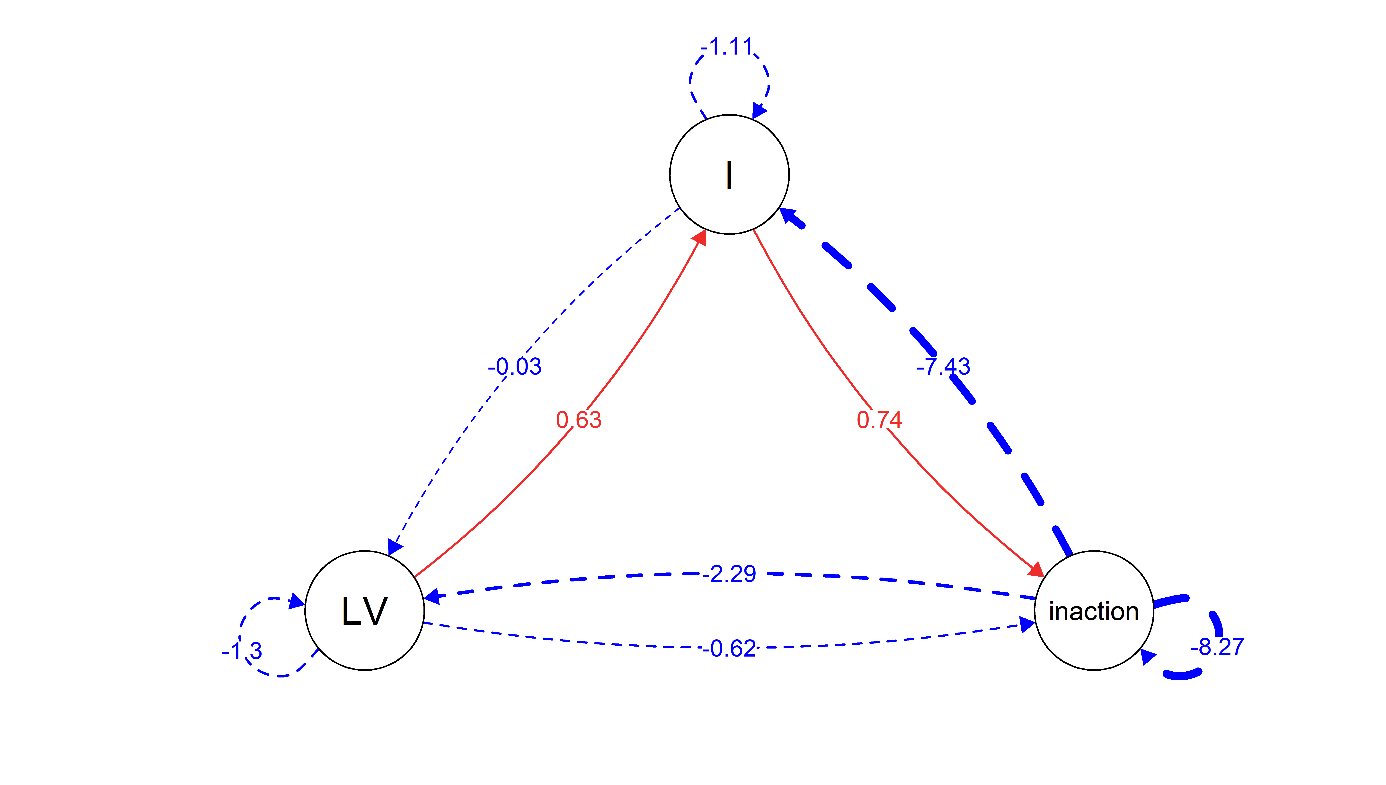


**Figure S5c.** Second iteration of least central network node for participant six


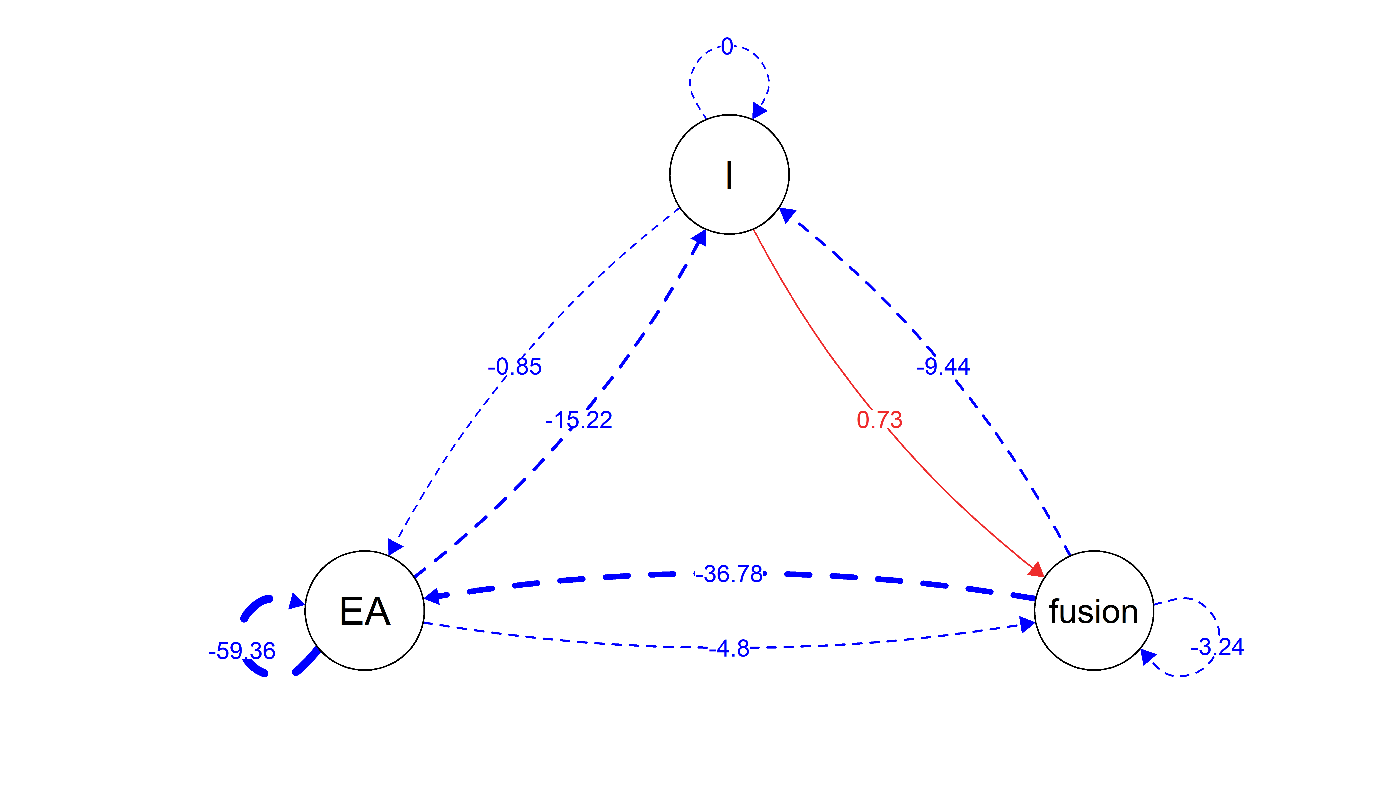


*Note.* I = Interference. O = Lack of openness. A = Lack of awareness. E = Lack of engagement. LV = Lack of values clarity. EA = Experiential avoidance.

**Table S1.** Exact total effect centrality values

*a) First iteration*

| ID | Interference | Open | Aware | Engaged |
| --- | --- | --- | --- | --- |
| 1 | 0.92 | -1.89 | 0.79 | 0.14 |
| 2 | -0.05 | -0.15 | 0.50 | 0.17 |
| 3 | 0.48 | -0.58 | 0.54 | -0.49 |
| 4 | 0.05 | 0.00 | 0.20 | -0.06 |
| 5 | -1.21 | 0.96 | -1.30 | 0.36 |
| 6 | -0.17 | -2.60 | 0.24 | 1.56 |

*Note*. Centrality values are calculated based on delta time corresponding to median time interval between assessments

*b) Second iteration*

|  | Second iteration of the most central node | | | Second iteration of the least central node | | |
| --- | --- | --- | --- | --- | --- | --- |
| ID | Interference | Most central | Least central | Interference | Most central | Least central |
| 1 | 0.07 | LPMA = 2.00 | SAC = -0.55 | 1.98 | EA = 0.55 | Fusion = -1.59 |
| 2 | -0.20 | SAC = 0.12 | LPMA = 0.05 | -0.03 | EA = 0.10 | Fusion = -0.15 |
| 3 | 0.13 | LPMA = -0.25 | SAC = -0.40 | 0.00 | Fusion = 0.29 | EA = -0.99 |
| 4 | -0.05 | LPMA = 0.53 | SAC = 0.12 | -0.65 | LV = -0.25 | Inaction = -0.36 |
| 5 | -0.16 | EA = 0.12 | Fusion = -0.54 | 0.06 | LPMA = 0.04 | SAC = -0.66 |
| 6 | 0.04 | LV = 0.05 | Inaction = -0.67 | 0.04 | EA = -0.26 | Fusion = -0.53 |

*Note.* Centrality values are calculated based on delta time corresponding to median time interval between assessments. LPMA = Lack of present moment awareness. SAC = Self as content. EA = Experiential avoidance. LV = Lack of values clarity.

**Table S2.** Most central overarching nodes at different posteriors

|  | | Most central | | Least central | |
| --- | --- | --- | --- | --- | --- |
| ID |  | TEC | IEC | TEC | IEC |
| 2 | 2.5 % | Lack of awareness | Lack of engagement | Lack of openness | Lack of awareness |
|  | 50 % | Lack of awareness | Lack of engagement | Lack of openness | Lack of awareness |
|  | 97.5 % | Lack of engagement | Lack of openness | Lack of openness | Lack of awareness |
|  |  |  |  |  |  |
| 3 | 2.5 % | Lack of openness | Lack of openness | Lack of awareness | Lack of engagement |
|  | 50 % | Lack of awareness | Lack of openness | Lack of openness | Lack of engagement |
|  | 97.5 % | Lack of awareness | Lack of engagement | Lack of openness | Lack of openness |
|  |  |  |  |  |  |
| 6 | 2.5 % | Lack of awareness | Lack of awareness | Lack of openness | Lack of engagement |
|  | 50 % | Lack of engagement | Lack of openness | Lack of openness | Lack of engagement |
|  | 97.5 % | Lack of engagement | Lack of openness | Lack of openness | Lack of awareness |

*Note.* TEC = Total effect centrality. IEC = Indirect effect centrality.
